# Influence of lipid vesicle properties on the function of conjugation dependent membrane active peptides†

**DOI:** 10.1039/d4tb01107d

**Published:** 2024-09-05

**Authors:** Alexandra Iversen, Johanna Utterström, Lalit Pramod Khare, Daniel Aili

**Affiliations:** a Laboratory of Molecular Materials, Division of Biophysics and Bioengineering, Department of Physics, Chemistry, and Biology, Linköping University 581 83 Linköping Sweden daniel.aili@liu.se

## Abstract

Membrane active peptides (MAPs) can provide novel means to trigger the release of liposome encapsulated drugs to improve the efficacy of liposomal drug delivery systems. Design of MAP-based release strategies requires possibilities to carefully tailor the interactions between the peptides and the lipid bilayer. Here we explore the influence of lipid vesicle properties on the function of conjugation-dependent MAPs, specifically focusing on two *de novo* designed peptides, JR2KC and CKV_4_. Utilizing liposomes with differences in size, lipid composition, and surface charge, we investigated the mechanisms and abilities of the peptides to induce controlled release of encapsulated cargo. Our findings indicate that liposome size modestly affects the structural changes and function of the peptides, with larger vesicles facilitating a minor increase in drug release efficiency due to higher peptide-to-liposome ratios. Notably, the introduction of negatively charged lipids significantly enhanced the release efficiency, predominantly through electrostatic interactions that favor peptide accumulation at the lipid bilayer interface and subsequent membrane disruption. The incorporation of cholesterol and a mix of saturated and unsaturated lipids was shown to alter the vesicle's phase behavior, thus modulating the membrane activity of the peptides. This was particularly evident in the cholesterol-enriched liposomes, where JR2KC induced lipid phase separation, markedly enhancing cargo release. Our results underscore the critical role of lipid vesicle composition in the design of MAP-based drug delivery systems, suggesting that precise tuning of lipid characteristics can significantly influence their performance.

## Abstract 2

Membrane active peptides (MAPs) can provide novel means to trigger the release of liposome encapsulated drugs to improve the efficacy of liposomal drug delivery systems.

## Graphical abstract

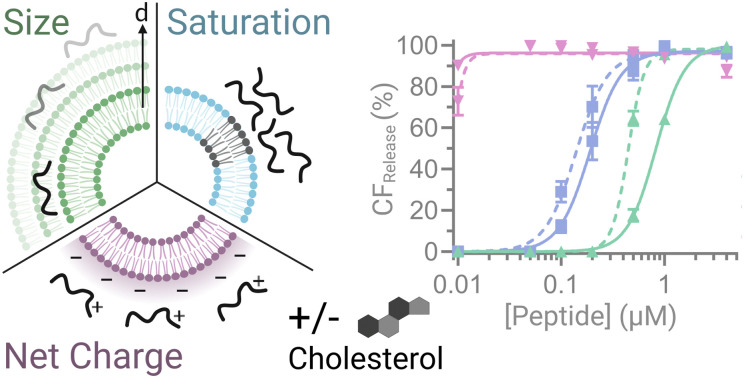

## Introduction

Liposomes are artificial lipid vesicles that are widely used in drug delivery applications. The first liposomal drug delivery system (DDS), Doxil®, was approved in 1995.^1^ Since then, several liposome-based drug formulations have been approved for the treatment of various cancers and fungal infections and as vaccines.^1,2^ Liposomes can enable efficient encapsulation of both hydrophilic and hydrophobic drug molecules, and demonstrate good biocompatibility, biodegradability, and long systemic circulation times. Encapsulation can improve the toxicity profile of the drug and improve drug accumulation in the targeted tissues. However, because of the slow release of the encapsulated compounds, the efficacy of the drug is typically not drastically improved.^3^ A slow release in the target tissue results in limited drug availability and prevents the drug from exerting its therapeutic effect. To solve this issue, numerous strategies to trigger release of the liposome content have been investigated,^4^ including irradiation of photoreactive groups,^5^ local heating caused by incorporated iron oxide nanoparticles when exposed to alternating magnetic fields,^6^ and liposome stabilizing moieties that can trigger membrane destabilization upon degradation by overexpressed extracellular enzymes.^7,8^ Despite significant research efforts, no such release mechanisms has been translated to clinical use.

Membrane active peptides (MAPs) offer interesting possibilities to modulate liposomal release due to their potential to selectively disrupt lipid membrane integrity. Several strategies to utilize MAPs for triggered release have been proposed where the MAP either intrinsically cause membrane destabilization^9–12^ or after protease activation.^13–15^ MAPs have a very large functional, chemical, and structural diversity, but most peptides used for drug delivery applications are short, cationic, and amphipathic, with sequences typically derived from, or inspired by, antimicrobial peptides (AMPs). Like AMPs, they often lack secondary structure in solution but fold into well-defined α-helices when interacting with lipid membranes. The peptide–membrane partitioning process involves both electrostatic and hydrophobic interactions making the MAP function highly dependent on the lipid properties of the vesicles. The close relationship between MAP function and lipid properties was clearly demonstrated by Sevcsik *et al.* where the mechanism of action of the three different MAPs; LL-37, melittin and PGLa, was shown to strongly depend on both lipid headgroup charge and hydrocarbon chain length.^16^ Cholesterol is often included as a lipid component in liposomes. Cholesterol alters membrane fluidity and phase behavior and can promote the formation of liquid-ordered (L_o_) phases. These L_o_ phases are distinct from the more fluid liquid-disordered (L_d_) phases, where unsaturated lipids predominate. Lipid vesicles containing cholesterol and a combination of saturated and unsaturated lipid species are thus prone to lipid-phase separation, which has been used for *e.g.*, tuning the avidity of liposome-bound ligands for cell targeting^17^ and to spatially control protein presentation on lipid vesicles to improve the cytotoxicity of therapeutic anticancer proteins.^18^ Lipid phase separation can also influence the function of MAPs. Antimicrobial peptide activity is often limited by a high cholesterol content^19^ but phase separation has been demonstrated to enhance the activity of some MAPs.^20,21^ Lipid phase separation can also be induced by peptide–lipid interactions mediated by MAPs, as a result of clustering of anionic lipids,^22^ or driven by more specific lipid-conjugation dependent and folding-mediated peptide–lipid interactions.^11^ The membrane activity of amphipathic peptides can be further enhanced by lipidation, *i.e.*, the attachment of one or more lipid groups to the peptides. Lipidation of AMPs has been widely exploited to improve their antibacterial activity.^23^

**Scheme 1:**
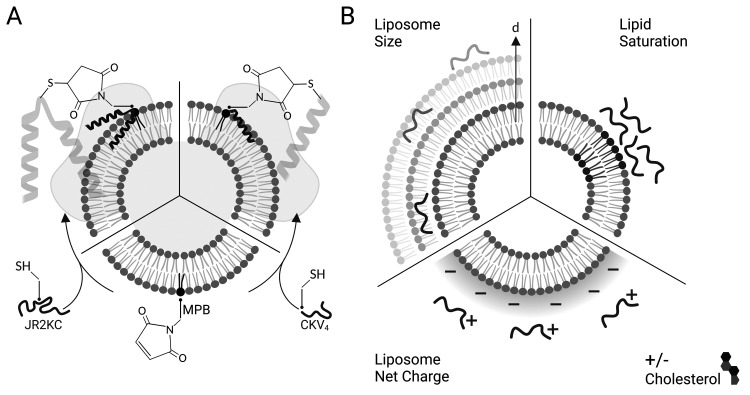
(A) The thiol-containing peptides JR2KC and CKV_4_ were conjugated to maleimide-functionalized liposomes *via* a Michael addition reaction, which triggered peptide folding and liposome cargo release. (B) The effect of liposome size (d), cholesterol, fraction of saturated lipids, and liposome net charge, on peptide-triggered release was explored.

We have previously investigated two different *de novo* designed MAPs; JR2KC and CKV_4_, that both selectively disrupt the integrity of POPC–lipid vesicles. The mechanisms involved in membrane disruption are not yet understood in detail but both peptides are conjugation-dependent and are thus not membrane active unless covalently conjugated to the outer leaflet *via* headgroup functionalized lipids (Scheme 1A). The need for conjugation is a result of the low intrinsic membrane affinity of the peptides. Conjugation results in *in situ* peptide lipidation and an accumulation of the peptides at the membrane interface, which lowers the barrier for peptide–lipid membrane partitioning. In addition, we have shown that the membrane activity of JR2KC and CKV_4_ is folding dependent and can be inhibited by introducing complementary peptides designed to heterodimerize with JR2KC and CKV_4_ and fold into a four-helix bundle and coiled coil, respectively.^9,13^ The membrane activity can be regained after heterodimer exchange^9^ or by proteolytic degradation of the complementary peptide.^13^ Additionally, peptide length was found to affect the activity of CKV_4_^10^ while cholesterol-rich lipid vesicles enhanced the activity of JR2KC due to a peptide-folding triggered lipid phase separation.^11^ The composition of the liposome must be carefully optimized to be relevant for drug delivery application to prevent premature drug release while evading the immune system long enough for accumulation in the target tissue. Common methods to improve stability of liposomes includes incorporation of cholesterol and/or saturated lipids in the lipid composition, reducing membrane fluidity and promoting drug retention.^24^ However, changes in lipid properties could influence the membrane destabilizing effect of the conjugated peptides and thus the possibilities to control and tune the drug release rate.

Here we explore how the physicochemical properties of liposomes, including size and lipid composition, affect the membrane activity of JR2KC and CKV_4_ (Scheme 1B). Despite some similarities in the design and function, the two peptides show large differences in molecular weight and net charge and thus responded differently to changes in liposome properties. Whereas the structure of the larger 42-residue peptide JR2KC was not influenced by liposome size, a decrease in liposomes size resulted in a significant increase in helicity for the shorter 29-residue peptide CKV_4_ upon lipid conjugation. Combining saturated and unsaturated lipid species resulted in a significant increase in the release rate of liposome encapsulated carboxyfluorescein (CF) for both JR2KC and CKV_4_, although liposome aggregation was observed for the former, possibly mediated by peptide-induced lipid phase separation. Increasing the negative net charge of the liposomes had a substantial effect on the peptide–lipid membrane interactions for both peptides and both peptides were seen to cause membrane disruption without being conjugated to the liposome surface. Reducing the membrane fluidity by incorporating cholesterol reversed the effect and the release process again became conjugation dependent. Increasing the liposome negative net charge reduced the colloidal stability of the liposomes when exposed to JR2KC but not CKV_4_. Moreover, JR2KC folded into a β-sheet like structure whereas CKV_4_ adopted an α-helical conformation on highly negatively charged liposomes, most likely due to the difference in number and distribution of positively charged lysine residues in the two peptides. These results demonstrate that MAPs with different net charge and molecular weight respond differently to changes in liposome properties and highlights the importance of optimization of both peptides and lipid vesicles in the design of MAP-mediated drug delivery systems for controlled release.

## Materials and methods

### General

All lipids were purchased from Avanti polar lipids (Alabaster), while chemicals were acquired from Sigma-Aldrich (Sigma-Aldrich, Saint Louis, Missouri) or Fischer Scientific (Hampton, New Hampshire, USA).

### Peptide synthesis

JR2KC and CKV_4_ were synthesized using Fmoc-chemistry on an automated microwave peptide synthesizer (Liberty Blue, CEM, Matthews, NC, USA) in 100 μM scale. Cl-MPA ProTide resin and ProTide Rinkamide (LL) resin were used as solid support for synthesis of JR2KC and CKV_4_ respectively. In case of JR2KC, the first amino acid was attached using a mixture of anhydrous KI and diisopropylethylamine (DIEA) in DMF under microwave conditions. For synthesis, Fmoc-protected amino acids were sequentially coupled using a four-fold excess of amino acids, Oxyma as base and DIC as coupling reagent in DMF under microwave conditions. Fmoc-deprotection was achieved by treatment with 20% piperidine in DMF under microwave conditions. After final Fmoc-deprotection N-terminal acetylation was performed on CKV_4_ by acetic anhydride and DIEA (5 eq.) in DMF for 1 h. Global deprotection and cleavage of peptides from resin was achieved by treatment with TFA : H_2_O : phenol : DTT (88/5/5/2, v/v/v/v) for 3 h before being concentrated using a stream of nitrogen. The crude peptides were precipitated in ice-cold diethyl ether, twice, and the ether was discarded. The crude peptides were purified on a semi-preparative HPLC system (dionex) equipped with a RP C-18 column (ReproSil Gold) using a gradient of acetonitrile containing 0.1% TFA. Peptide identity and purity was confirmed using MALDI-ToF mass spectrometer (Bruker) and HPLC (Thermofischer), respectively.

### Vesicle preparation

Large unilamellar vesicles were prepared by thin-film hydration followed by extrusion. Lipids dissolved in chloroform were mixed in desired mol% followed by evaporation of the solvent using a stream of nitrogen. Residual chloroform was removed by placing the lipid films in a vacuum desiccator overnight. The next day, the lipid films were rehydrated by 10 min incubation in buffer followed by 1 min vortex, generating a total lipid concentration of 5 mg ml^−1^. Vesicle dispersion was reduced with a mini extruder (Avanti polar lipids, Alabaster, Alabama) by extruding the lipid suspension 21 times through a 50, 100 or 200 nm polycarbonate membrane. 10 mM phosphate buffer (PB), pH 7.4, was used to rehydrate the lipid films for CD measurements while 10 mM PBS (140 mM sodium chloride, 2.7 mM potassium chloride, and 10 mM phosphate), pH 7.4, was used for the FRET assay. CF release assays and DLS measurements were instead prepared by rehydrating the vesicles in 50 mM carboxyfluorescein (CF, self-quenching concentration) in 10 mM PB with 90 mM NaCl, pH 7.4. Unencapsulated CF was removed by size exclusion chromatography, using a G-25 column (Cytiva, Marlborough, Massachusetts, USA) equilibrated with PBS (10 mM, pH 7.4), prior the CF release assay.

### Carboxyfluorescein (CF) release assay

Peptide triggered release from lipid vesicles was studied on a fluorescence plate reader (Tecan Infinite M1000 Pro, Tecan Austria GmbH, Grödig/Salzburg, Austria) by monitoring the CF-fluorescence. Encapsulated at self-quenching concentrations, release of CF will increase the fluorescence signal (*λ*_ex_ = 485 nm and *λ*_em_ = 520 nm) when measured over time. Initially, the background fluorescence from merely vesicles (*F*_0_) in PBS (10 mM, pH 7.4) was measured before the kinetic run was started by adding the peptide, or PBS buffer for the blank, to a final lipid concentration of 40 μM. The fluorescence (*F*_*t*_) was measured during 30 min for JR2KC (0.01, 0.05, 0.1, 0.2, 0.5, 1 and 4 μM) and 2 h for CKV_4_ (0.1, 0.2, 0.5, 1, 2, 5 and 10 μM). Total vesicle lysis was achieved by incubating the vesicles with Triton X-100 (0.1%) for 10 min before measuring the fluorescence once again (*F*_tot_). Total CF release at a certain time point was calculated using 
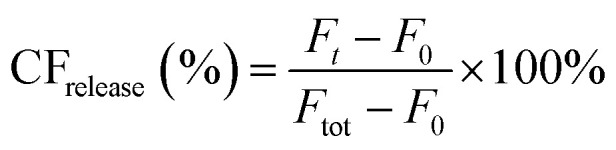
 and these values at the end of each kinetic run were plotted against the peptide concentration. A Hill equation 
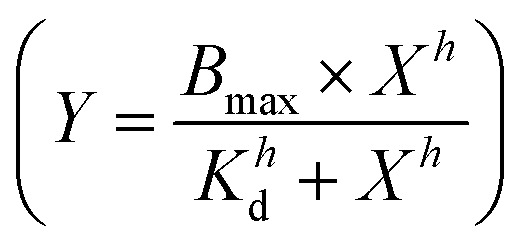
 was then used to fit the plotted total CF release data, where *B*_max_ is the maximum CF release, *X* is the peptide concentration, *h* is the Hill coefficient and *K*_d_ is the peptide concentration required to achieve half of the maximum CF release.

### Circular dichroism (CD)

All CD measurements were performed on a Chirascan (applied photophysics, Leatherhead, United Kingdom) in room temperature using a 1 mm pathlength quartz cuvette. The scanning was performed between 200 and 280 nm with 0.5 nm step length. All samples were background subtracted using pure PB for the peptide samples while vesicles in PB were used for peptides conjugated to vesicles. Peptide concentration was 30 μM in all measurements and when measured together with vesicles the peptide : MPB ratio was 1 : 2, corresponding to a total lipid concentration of 1.2 mM. Peptide–lipid samples were incubated for 30 min (JR2KC) and 2 h (CKV_4_) respectively before measuring. Background measurements were recorded three times while all peptide-containing samples were recorded five times. These were then averaged and smoothed using the Savitzky–Golay algorithm.

### Dynamic light scattering (DLS)

An ALV/DLS/SLS-5022F system from ALV-GmbH (Langen, Germany) equipped with temperature control and a 632.8 nm HeNE laser was used to determine the size distribution of the vesicles. The temperature was set to 22 °C and all samples were prepared in filtered (0.22 μm) PBS (10 mM, pH 7.4) with a lipid concentration of 40 μM. Peptide concentrations were chosen to match the highest peptide concentration used in the CF release assay, thus 4 μM JR2KC and 10 μM CKV_4_. The correlation curves were obtained by averaging 10 consecutive 30 s runs and the vesicle size distribution was calculated by the CONTIN 2DP routine implemented in the ALV software.

### Zeta potential

The *ζ*-potential was measured on a Malvern ZetaSizer Nano ZS90 (Malvern Panalytical, Malvern, Worcestershire, United Kingdom). The samples were prepared with a lipid concentration of 650 μM in PB (10 mM, pH 7.4).

### Fluorescence resonance energy transfer (FRET)

The lipid conjugated FRET pair 1,2-dipalmitoyl-*sn-glycero*-3-phosphoethanolamine-*N*-(7-nitro-2-1,3-benzoxadiazol-4-yl) (16 : 0 NBD, donor) and 1,2-dioleoyl-*sn-glycero*-3-phosphoethanolamine-*N*-(lissamine rhodamine B sulfonyl) (18 : 1 Rhod, acceptor) were added (0.5 mol%) to each lipid composition during lipid film preparation. Vesicles were prepared in PBS (10 mM, pH 7.4) at a concentration of 40 μM and then the fluorescence emission of the donor and acceptor were measured on a fluoromax-4 spectrophotometer (Horiba Jobin Yvon Inc., United States) at 535 nm and 583 nm respectively after exciting the NBD-fluorophore at 460 nm. 4 μM JR2KC or 10 μM CKV_4_ were then added, corresponding to the highest concentration used in the CF release assay, and the fluorescence emission was measured again after 30 min (JR2KC) and 2 h (CKV_4_) incubation respectively. The normalized FRET ratio was calculated for solely vesicles or peptide conjugated vesicles using: 
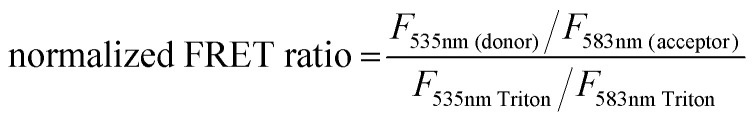
, where *F*_Triton_ is the fluorescence emission at 535 nm and 583 nm after incubating the vesicles with Triton X-100 (0.1%) for 10 min. All samples were recorded in triplicates.

## Results and discussion

### Peptide design

JR2KC and CKV_4_ are two *de novo* designed conjugation- and folding-dependent membrane active peptides,^9–11,13^ that are unstructured in solution at neutral pH but fold into well-defined α-helices when covalently conjugated to 1-palmitoyl-2-oleoyl-*glycero*-3-phosphocholine (POPC) liposomes comprising 5 mol% of the maleimide functionalized lipid (1,2-dioleoyl-*sn-glycero*-3-phosphoethanolamine-*N*-[4-(*p*-maleimidophenyl)butyramide] (MPB-PE)) *via* a Michael addition reaction utilizing a single cysteine (Cys) residue in the peptides. JR2KC and CKV_4_ are both amphipathic and have lysine (Lys, K) rich primary sequences, rendering them a net positive charge of +11 and +4, respectively, at physiological pH (Table 1). JR2KC is composed of 42 amino acids and designed to fold into a helix-loop-helix motif and have a Cys residue in the loop-region (position 22) for lipid conjugation. CKV_4_ is shorter, with 29 amino acids and four identical heptad repeats, and is designed to fold into a single helix when conjugated to a lipid membrane *via* a N-terminal Cys. Both peptides are designed to dimerize, either as homodimers at basic pH values, or as heterodimers with charge complementary peptides at neutral pH. Whereas JR2KC folds into a four-helix bundle upon dimerization, CKV_4_ forms a parallel coiled coil.

**Table 1:** Primary sequence, molecular weight, and net charge at pH 7 of JR2KC and CKV_4_

Name	Primary sequence	MW (Da)	Net charge at pH 7
JR2KC	H_2_N-NAADLEKAIEALEKHLEAKGPCDAAQLEKQLEQAFEAFERAG-COOH	4581.28	+11
CKV_4_	Ac-CKVSALKEKVSALKEKVSALKEKVSALKE-NH_2_	3183.86	+4

### Effect of liposome size on conjugation dependent membrane activity

**Fig. 1:**
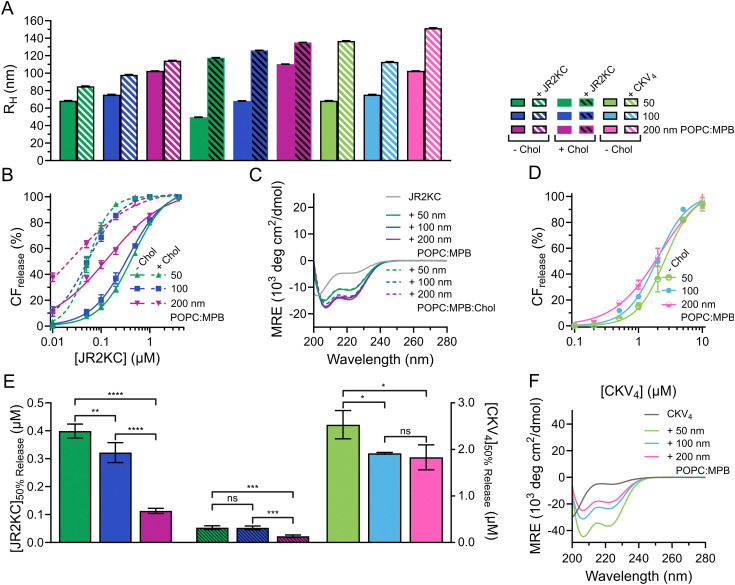
(A) Hydrodynamic radius of liposomes (50, 100, 200 nm, 40 μM) before and after incubation with JR2KC (0.5 hours, 4 μM) or CKV_4_, (2 hours, 10 μM). (B) Total CF release from 95 : 5 (solid) and 65 : 5 : 30 (dashed) POPC : MPB : Chol liposomes (40 μM) with varying size (50, 100, 200 nm) after 30 min incubation with JR2KC. Data was fitted to a Hill equation, *N* = 3. (C) CD spectra of 30 μM JR2KC alone and after 30 min incubation with 1.2 mM 95 : 5 and 65 : 5 : 30 POPC : MPB : Chol liposomes of varying hydrodynamic radius (50, 100, 200 nm). (D) Total CF release from 40 μM 95 : 5 POPC : MPB liposomes with varying hydrodynamic radius (50, 100, 200 nm) after 2 hours incubation with CKV_4_. Data was fitted to a Hill equation, *N* = 3. (E) Estimated peptide concentration required to reach 50% CF release from liposomes with varying hydrodynamic radius (50, 100, 200 nm) after 30 min incubation with JR2KC or 2 hours incubation with CKV_4_ respectively, based on the fittings in (B) and (D). (F) CD spectra of 30 μM CKV_4_ alone and after 2 hours incubation with 1.2 mM 95 : 5 POPC : MPB liposomes of varying hydrodynamic radius (50, 100, 200 nm).

The diameter (d) of most commercial liposomal DDSs is ≤110 nm^25^ to take advantage of the enhanced permeability and retention effect caused by the leaky vasculature in the tumor tissues. In tumors, the fenestrations or endothelial gaps between endothelial cells lining the capillaries are usually between 100 nm to 2 μm.^26^ Many MAPs have shown preference for regions of higher curvature and can thus be influenced by liposome size.^27^ To explore the effect of liposome size on the membrane activity of JR2KC and CKV_4_, lipid vesicles composed of 95 : 5 POPC : MPB (MPB liposomes) and 65 : 5 : 30 POPC : MPB : Chol (MPB/Chol liposomes) were prepared by thin film hydration and then subjected to extrusion through polycarbonate (PC) membranes with defined pore sizes of 50, 100 and 200 nm, respectively. Small unilamellar vesicles (SUV), <100 nm in diameter, are preferably formed using sonication^28^ but to keep the preparation method the same, extrusion was used for all liposomes. The hydrodynamic radius (*R*_H_) of the obtained MPB and MPB/Chol liposomes were 68, 75, 103 nm, and 50, 68, and 110 nm, respectively, for the three different PC pore sizes (Fig. 1A and Fig. S1, ESI†). Since the difference in hydrodynamic radius also varied slightly for the different lipid compositions, we will refer to the size of the liposomes based on the pore size of the PC membranes used.

To study the lipid membrane activity of JR2KC and CKV_4_, we encapsulated self-quenching concentrations (50 mM) of carboxyfluorescein (CF) in the liposomes. The CF release was then monitored over time. No CF release was obtained in the absence of peptides, or in the absence of MPB (Fig. S2 and S3, ESI†). Due to the relatively small difference in liposomes size, and hence curvature, no significant difference in release kinetics was observed for the 50 and 100 nm MPB liposomes when incubated with JR2KC (Fig. 1B and Fig. S4A, B, ESI†). However, the activity of JR2KC on the larger, 200 nm, MPB-liposomes resulted in an increase in both the release kinetics and extent of the release (Fig. 1B and Fig. S4C, ESI†). The release mechanism of JR2KC has previously been determined to be folding dependent^11,13^ but CD measurements indicated only minor increase in helicity with increasing liposomes size (Fig. 1C and Table 2). Moreover, JR2KC did not trigger aggregation of any of the liposomes (Fig. 1A and Fig. S1A, ESI†). The increase in CF release seen for 200 nm MPB-liposomes could be explained by the constant lipid concentration used in all experiments (40 μM), which gives the same peptide/lipid ratio in all cases. Thus, when increasing liposome size, more peptides will be bound to each liposome.^29^ We have previously observed that a threshold concentration of conjugated JR2KC is needed to trigger CF release.^13^ Thus, it was not surprising that JR2KC caused more efficient release in 200 nm MPB-liposomes compared to 50 and 100 nm vesicles. For CKV_4_, the effect of increasing liposomes was less pronounced with respect to CF release (Fig. 1D and Fig. S4D–F, ESI†). Although a small increase in total CF release after 2 h was observed for the lower peptide concentrations on 200 nm MPB-liposomes, the peptide concentration required to reach 50% CF release after 2 h incubation ([peptide]_50% CF release_) was the same as for 100 nm MPB-liposomes (Fig. 1E). However, CKV_4_ showed a large increase in helicity with decreasing liposomes size, which likely can be attributed to the increase in curvature (Fig. 1F and Table 2). In addition, CKV_4_ induced slightly larger changes in *R*_H_ than JR2KC (Fig. 1A and Fig. S1B, ESI†), which could be due to higher concentrations of accumulated peptides on the liposomes as more CKV_4_ was required to trigger efficient CF release compared to JR2KC.

**Table 2:** Hydrodynamic radius, MRE_222_ and ratio of MRE at 222 and 208 nm for JR2KC and CKV_4_ alone and after incubation with liposomes of varying size (50, 100, 200 nm)

Peptide	Liposome	Size (nm)	*R*_H_ (nm)	MRE_222_ (×10^3^) (deg cm^2^ dmol^−1^)	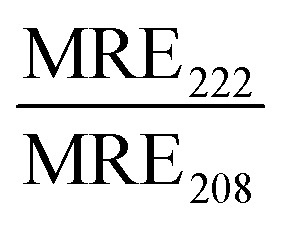
—	POPC:MPB	50	68	—	—
		100	75	—	—
		200	103	—	—
	POPC:MPB:Chol	50	50	—	—
		100	68	—	—
		200	110	—	—

JR2KC	—	—	—	−4.8	0.5
	POPC:MPB	50	85	−11.3	0.8
		100	98	−13.9	0.8
		200	114	−14.7	0.8
	POPC:MPB:Chol	50	118	−10.8	0.7
		100	126	−14.0	0.8
		200	135	−13.4	0.8

CKV_4_	—	—	—	−5.4	0.4
	POPC:MPB	50	137	−37.1	0.8
		100	113	−23.7	0.8
		200	152	−19.0	0.8

JR2KC has previously been found to induce a folding-dependent lipid phase separation in cholesterol (Chol) containing liposomes. In MPB liposomes with 30 mol% Chol, the clustering of MPB–lipids upon JR2KC conjugation greatly enhanced the CF release.^11^ When including cholesterol in the liposome composition for the three different sizes, the same trends were seen as for non-cholesterol liposomes, with respect to the increase in CF release (Fig. 1B and Fig. S4G–I, ESI†), α-helical content (Fig. 1C and Table 2) and liposome size increase after peptide addition (Fig. 1A and Fig. S1C, ESI†).

### Influence of saturated lipids on the membrane activity of JR2KC and CKV_4_

In liposomal DDSs, lipid saturation significantly influences drug retention and release.^30^ Saturated lipids generally enhance retention by stabilizing the membrane but can restrict drug release due to reduced membrane fluidity. In contrast, unsaturated lipids increase membrane fluidity, promoting quicker and more efficient drug release, though potentially at the cost of reduced structural stability and increased susceptibility to oxidation.^31^ Including Chol in liposomes composed of both saturated and unsaturated lipids enhances the structural ordering of the membrane and reduces its overall fluidity, which stabilizes the lipid bilayer across a range of temperatures and reduces susceptibility to phase transitions. The incorporation of cholesterol also promote the formation of distinct lipid domains, which previously have been shown to enhance the membrane activity of JR2KC.^11^

**Fig. 2:**
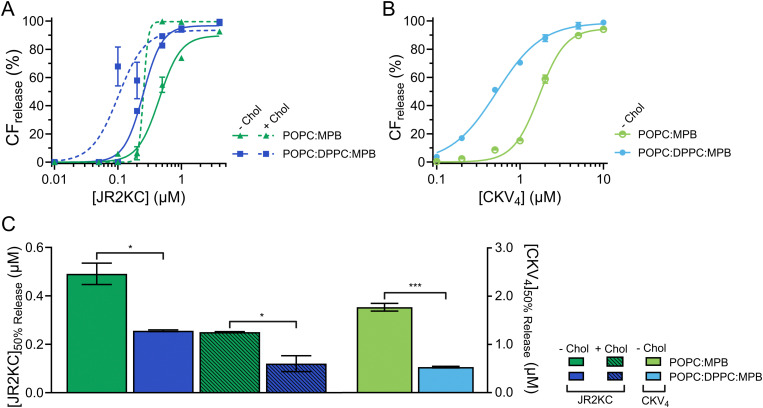
(A) Total CF release from 40 μM 95 : 0 : 5 : 0, 65 : 0 : 5 : 30, 47.5 : 47.5 : 5 : 0, and 32.5 : 32.5 : 5 : 30 POPC : DPPC : MPB : Chol liposomes with varying mol% lipids with saturated acyl chains after 30 min incubation with JR2KC. Data was fitted to a Hill equation, *N* = 3. (B) Total CF release from 40 μM 95 : 0 : 5, and 47.5 : 47.5 : 5 POPC : DPPC : MPB liposomes with varying mol% lipids with saturated acyl chains after 2 hours incubation with CKV_4_. Data was fitted to a Hill equation, *N* = 3. (C) Estimated peptide concentration required to reach 50% CF release from liposomes with varying mol% lipids with saturated acyl chains after 30 min incubation with JR2KC or 2 hours incubation with CKV_4_ respectively, based on the fittings in (A) and (B).

To assess the effect of saturated lipids on the membrane activity of JR2KC and CKV_4_, 1,2-dipalmitoyl-*sn-glycero*-3-phosphocholine (DPPC) was included at a 1 : 1 POPC : DPPC ratio, both with and without cholesterol, generating 47.5 : 47.5 : 5 mol% POPC : DPPC : MPB and 32.5 : 32.5 : 5 : 30 mol% POPC : DPPC : MPB : Chol liposomes. Liposomes without cholesterol but including DPPC showed a slight increase in the zeta potential (Δ*ζ* = +6.2 mV) compared to POPC : MPB liposomes (Fig. S5, ESI†). However, this difference was not seen for liposomes with both cholesterol and DPPC (Fig. S5, ESI†). All DPPC-containing liposomes were stable, and no CF release was seen in the absence of peptides or in the presence of peptides but absence of MPB (Fig. S6 and S7, ESI†). However, addition of peptides to MPB containing liposomes resulted in a significantly faster and more extensive CF release from liposomes with DPPC compared to without DPPC for both JR2KC (Fig. 2A and Fig. S8A, B, ESI†) and CKV_4_ (Fig. 2B and Fig. S8C, D, ESI†). The decrease in [peptide]_50% CF release_ was approximately two-fold for JR2KC, both with and without cholesterol, and more than three-fold for CKV_4_ (Fig. 2C), compared to POPC:MPB liposomes. The increase in zeta potential for non-cholesterol containing liposomes with DPPC did consequently not have any negative effects on the CF release rate. Rather, the combination of saturated and unsaturated lipids promoted the interactions of the peptides with the liposomes resulting in more pronounced membrane destabilization. In contrast, the interaction of antimicrobial peptides, such as LL-37 and G4, tends to be less disruptive in lipid bilayers with saturated lipids compared to lipid membranes composed of unsaturated lipids.^32,33^

**Fig. 3:**
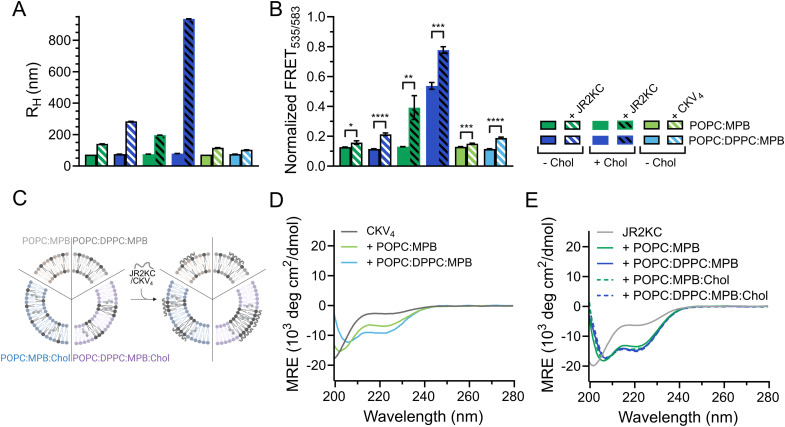
(A) Hydrodynamic radius of 40 μM liposomes with varying mol% lipids with saturated acyl chains before and after 30 min incubation with 4 μM JR2KC or 2 hours incubation with 10 μM CKV_4_ respectively. (B) Normalized FRET ratio (535 nm/583 nm) of 40 μM liposomes with varying mol% lipids with saturated acyl chains, with and without cholesterol, before and after incubation with 4 μM JR2KC for 30 min or 10 μM CKV_4_ for 2 hours. (C) No phase separation was seen in POPC:MPB or POPC:DPPC:MPB liposomes in neither absence nor presence of JR2KC or CKV_4_. JR2KC triggered a distinct phase separation in POPC:MPB:Chol and POPC:DPPC:MPB:Chol liposomes. (D) CD spectra of 30 μM CKV_4_ alone and after 2 hours incubation with 1.2 mM 95 : 0 : 5 or 47.5 : 47.5 : 5 POPC : DPPC : MPB liposomes with varying mol% lipids with saturated acyl chains. (E) CD spectra of 30 μM JR2KC alone and after 30 min incubation with 1.2 mM 95 : 0 : 5 : 0, 47.5 : 47.5 : 5 : 0, 65 : 0 : 5 : 30 or 32.5 : 32.5 : 5 : 30 POPC : DPPC : MPB : Chol liposomes with varying mol% lipids with saturated acyl chains.

DLS was utilized to explore the effect of peptide–lipid interactions on the size of the liposomes containing DPPC. A clear increase in *R*_H_ from about 76 to 197 nm was seen upon addition of JR2KC to POPC:MPB:Chol and from 76 to 284 nm upon addition to POPC:DPPC:MPB liposomes (Fig. 3A and Fig. S9A, B, ESI†). This could be an effect of peptides accumulating at the liposome surface, liposome swelling due to peptide insertion, or minor liposome aggregation. In contrast, addition of JR2KC to POPC:DPPC:MPB:Chol liposomes resulted in extensive aggregation, seen as an increase in *R*_H_ of 79 to >900 nm (Fig. 3A and Fig. S9B, ESI†). Surprisingly though, very small differences in *R*_H_ were seen before and after addition of CKV_4_ to DPPC-containing liposomes lacking cholesterol (Fig. 3A and Fig. S9C, ESI†). This could potentially be attributed to both differences in net charge and size of the two peptides in combination with differences in lipid-phase separation.

To investigate if the increase in CF release from DPPC-containing liposomes was influenced by lipid phase separation, 0.5 mol% of each of the fluorescence resonance energy transfer (FRET) pair 1,2-dipalmitoyl-*sn-glycero*-3-phosphoethanolamine-*N*-(7-nitro-2-1,3-benzoxadiazol-4-yl) (NBD, donor) and 1,2-dioleoyl-*sn-glycero*-3-phosphoethanolamine-*N*-(lissamine rhodamine B sulfonyl) (Rhod, acceptor) was included in the lipid compositions. The FRET ratio of POPC : MPB, POPC : DPPC : MPB and POPC : MPB : Chol liposomes were close to identical (0.1) in the absence of peptides, indicating that the combination of POPC and DPPC was not sufficient to trigger lipid phase separation (Fig. 3B, C, Table 3 and Fig. S10A–C, ESI†). However, the FRET ratio was significantly higher (0.5) for vesicles containing both cholesterol and a mixture of saturated and unsaturated lipid species (POPC:DPPC:MPB:Chol), clearly indicating that this lipid combination resulted in formation of defined lipid domains (Fig. 3B, C, Table 3 and Fig. S10D, ESI†). Addition of JR2KC or CKV_4_ to POPC:DPPC:MPB liposomes, resulted in a small increase in the FRET ratio from about 0.1 to 0.2. This could indicate that the peptides induced formation of smaller lipid domains, likely triggered by the electrostatic interaction between the positively charged peptide and the negatively charged MPB. Lipid bilayers containing saturated phospholipids typically show enhanced permeability in the temperature region of phase separation due to defects that develop at the boundaries between liquid crystal and gel state domains.^34^ The increase in CF release seen for the POPC:DPPC:MPB liposomes could thus be a result of peptide-induced lipid membrane phase separation.

**Table 3:** MRE at 222 nm and ratio of MRE at 222 nm and 208 nm for JR2KC alone and after 30 min incubation with liposomes with varying mol% lipids with saturated acyl chains, with and without cholesterol and for CKV_4_ after 2 hours incubation with liposomes with varying mol% lipids with saturated acyl chains, and FRET ratio of liposomes with varying mol% lipids with saturated acyl chains and with and without cholesterol alone and after 30 min incubation with JR2KC or 2 hours incubation with CKV_4_ respectively

Peptide	Liposome	MRE_222_ (×10^3^) (deg cm^2^ dmol^−1^)	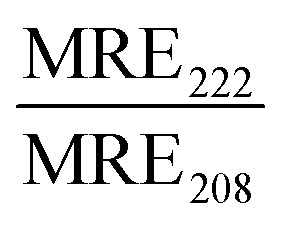	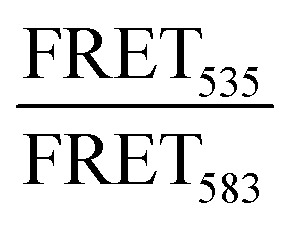
—	POPC:MPB	—	—	0.1
	POPC:DPPC:MPB	—	—	0.1
	POPC:MPB:Chol	—	—	0.1
	POPC:DPPC:MPB:Chol	—	—	0.5

JR2KC	—	−6.3	0.5	—
	POPC:MPB	−13.5	0.8	0.2
	POPC:DPPC:MPB	−14.6	0.8	0.2
	POPC:MPB:Chol	−14.4	0.8	0.4
	POPC:DPPC:MPB:Chol	−15.2	0.9	0.8

CKV_4_	—	−2.9	0.4	—
	POPC:MPB	−6.9	0.6	0.2
	POPC:DPPC:MPB	−9.4	0.8	0.2

The membrane activity of both CKV_4_ and JR2KC has previously been shown to be folding dependent. Here, the helicity of CKV_4_, increased when including DPPC in the lipid mixture (Fig. 3D and Table 3). In contrast, the secondary structure of JR2KC was similar for all lipid compositions, both with and without DPPC and/or cholesterol (Fig. 3E and Table 3). However, in liposomes containing cholesterol, JR2KC triggered a significant increase in the FRET ratio, both with and without DPPC, from 0.1 to 0.4 for POPC : MPB : Chol liposomes and from 0.5 to 0.8 for POPC : DPPC : MPB : Chol, clearly demonstrating that the conjugation and folding of the peptide can contribute to lipid phase separation (Fig. 3B, C, Table 3 and Fig. S10, ESI†), which is in line with our previous observations.^11^ Interestingly, also in POPC:DPPC:MPB:Chol liposomes with preformed lipid domains, additional lipid phase separation was induced upon addition of JR2KC. The formation of larger domains with high peptide surface concentrations, and thus high net charge, likely contributed to the observed aggregation of the liposomes. Aggregation could lead to fusion of the liposomes and could likely be reduced or prevented by introducing PEGylated lipids.

### Promoting peptide–liposome interactions by increasing vesicle net charge

The initial accumulation of membrane active peptides on lipid bilayers is primarily driven by electrostatic interactions.^35^ At the liposomes surface, structural rearrangements result in clustering of hydrophobic residues promoting hydrophobic interactions between the lipid acyl chains and hydrophobic amino acids in the peptide. Thus, modulating the liposome net charge could greatly influence the rate of peptide–membrane adsorption, which potentially will affect their activity. To investigate this effect, the negatively charged lipid 1-palmitoyl-2-oleoyl-*sn-glycero*-3-phospho-(1′-*rac*-glycerol) (POPG) was included in the lipid composition, at either 1 : 1 POPC : POPG ratio or by replacing all POPC lipids with POPG. This generated the liposomes 47.5 : 47.5 : 5 POPC : POPG : MPB; 95 : 5 POPG : MPB; 32.5 : 32.5 : 5 : 30 POPC : POPG : MPB : Chol; and 65 : 5 : 30 POPG : MPB : Chol.

**Fig. 4:**
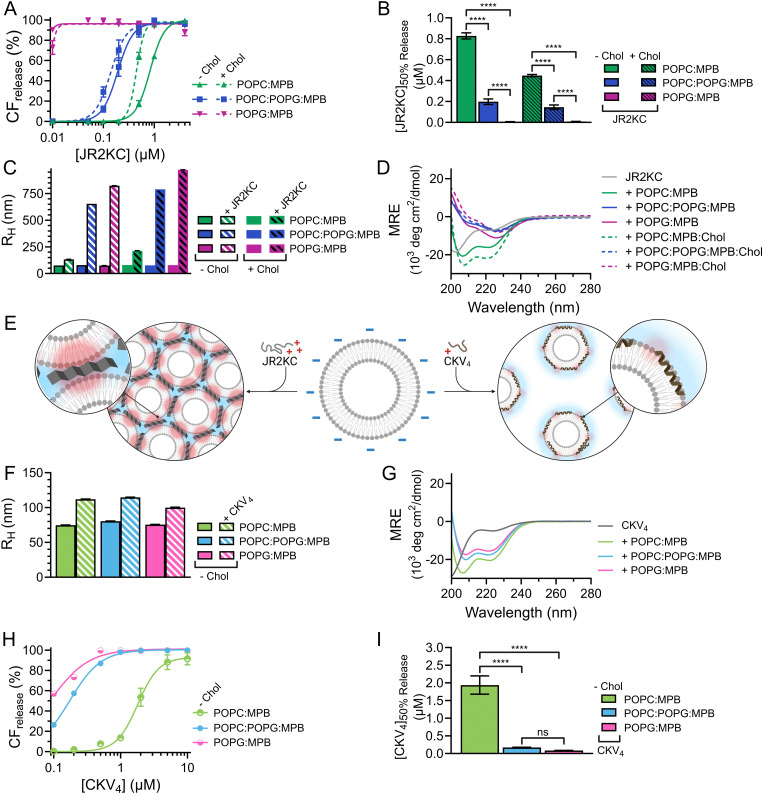
(A) Total CF release from 40 μM 95 : 0 : 5 : 0, 65 : 0 : 5 : 30, 47.5 : 47.5 : 5 : 0, 32.5 : 32.5 : 5 : 30, 0 : 95 : 5 : 0, and 0 : 65 : 5 : 30 POPC : POPG : MPB : Chol liposomes with varying mol% lipids with negatively charged headgroup after 30 min incubation with JR2KC. Data was fitted to a Hill equation, *N* = 3. (B) Estimated peptide concentration required to reach 50% CF release from liposomes with varying mol% lipids with negatively charged headgroup after 30 min incubation with JR2KC based on the fittings in (A). (C) Hydrodynamic radius of 40 μM liposomes with varying mol% lipids with negatively charged headgroup before and after 30 min incubation with 4 μM JR2KC. (D) CD measurements of 30 μM JR2KC alone and after 30 min incubation with 1.2 mM 95 : 0 : 5 : 0, 47.5 : 47.5 : 5 : 0, 0 : 95 : 5 : 0, 65 : 0 : 5 : 30, 32.5 : 32.5 : 5 : 30, or 0 : 65 : 5 : 30 POPC : POPG : MPB : Chol liposomes with varying mol% lipids with negatively charged headgroup. (E) Illustration showing aggregation of negatively charged POPG-containing liposomes in presence of JR2KC and its β-sheet structure meanwhile no aggregation occurs when these negatively charged liposomes are incubated with the less positively charged CKV_4_ that folds into an α-helix. (F) Hydrodynamic radius of 40 μM liposomes with varying mol% lipids with negatively charged headgroup before and after 2 hours incubation with 10 μM CKV_4_. (G) CD spectra of 30 μM CKV_4_ alone and after 2 hours incubation with 1.2 mM 95 : 0 : 5, 47.5 : 47.5 : 5, or 0 : 95 : 5 POPC : POPG : MPB liposomes with varying mol% lipids with negatively charged headgroup. (H) Total CF release from 40 μM 95 : 0 : 5, 47.5 : 47.5 : 5, 0 : 95 : 5 POPC : POPG : MPB liposomes with varying mol% lipids with negatively charged headgroup after 2 hours incubation with CKV_4_. Data was fitted to a Hill equation, *N* = 3. (I) Estimated peptide concentration required to reach 50% CF release from liposomes with varying mol% lipids with negatively charged headgroup after 2 hours incubation with CKV_4_ based on the fittings in (H).

As expected, when including high amounts of POPG in the liposomes, their zeta potential dropped drastically from −33.6 mV for POPC:MPB to −74.6 mV for POPC:POPG:MPB liposomes (Fig. S5, ESI†). Interestingly, there was no significant difference in zeta potential between POPC:POPG:MPB and POPG:MPB liposomes, neither with nor without cholesterol, although the latter contained higher quantities of negatively charged lipids. However, the extent and rate of CF release was greatly enhanced when incubating JR2KC with liposomes with increasing amount of POPG (Fig. 4A and Fig. S11A–C, ESI†). The CF release process was still peptide-dependent since no CF release was observed for any POPG-containing liposomes in the absence of peptides (Fig. S12, ESI†). For POPC:POPG:MPB liposomes, [JR2KC]_50% CF release_ decreased more than four-fold compared to POPC:MPB liposomes (Fig. 4B), clearly demonstrating that an increased electrostatic attraction between the peptide and liposomes accelerated peptide lipidation and the resulting peptide–membrane partitioning process. Additionally, completely exchanging all POPC for POPG lipids resulted in extremely efficient CF release, where 90% release was reached after just 30 min incubation with only 0.01 μM JR2KC. However, addition of JR2KC to POPG-containing lipid vesicles induced major liposome aggregation (Fig. 4C and Fig. S13A, ESI†). Additionally, CD measurements showed that JR2KC in combination with POPG-containing liposomes did not fold into an α-helix but instead adopted a clear β-sheet structure and thus a distinctly different organization of the peptides on the POPG-rich lipid membranes (Fig. 4D, E and Table 4).

**Table 4:** MRE at 222 nm and ratio between MRE at 222 and 208 nm for JR2KC alone and after 30 min incubation with liposomes with varying mol% POPG, with and without cholesterol, and for CKV_4_ after 2 hours incubation with liposomes with varying mol% POPG

Peptide	Liposome	MRE_222_ (×10^3^) (deg cm^2^ dmol^−1^)	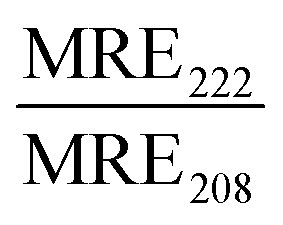
JR2KC	—	−7.0	0.5
	POPC:MPB	−16.3	0.8
	POPC:POPG:MPB	−6.7	2.7
	POPG:MPB	−10.4	2.8
	POPC:MPB:Chol	−21.9	0.9
	POPC:POPG:MPB:Chol	−5.7	−313.1
	POPG:MPB:Chol	−6.8	−4.5

CKV_4_	—	−5.2	0.4
	POPC:MPB	−20.5	0.7
	POPC:POPG:MPB	−17.4	0.9
	POPG:MPB	−15.9	0.9

In contrast, CKV_4_ did not reduce the colloidal stability of POPG-containing liposomes and the peptide adopted an α-helical conformation (Fig. 4E–G, Fig. S13B and Table 4, ESI†). Conjugation of CKV_4_ resulted in a massive increase in the CF release rate and the [CKV_4_]_50% CF release_ decreased with more than one order of magnitude for POPC:POPG:MPB liposomes compared to POPC:MPB liposomes (Fig. 4H, I and Fig. S11D–F, ESI†). The relative change in [CKV_4_]_50% CF release_ was hence a factor two higher than for JR2KC for the same lipid composition. Likely, the observed differences were a consequence of the differences in peptide net charge and size, where JR2KC is both a longer polypeptide and has a much higher positive net charge than CKV_4_. This means that JR2KC can contribute to both screening of liposome surface charge and trigger a bridging aggregation of the liposomes. Additionally, due to the high number of Lys residues in JR2KC, the peptide will likely adopt an elongated, β-sheet like conformation, since the electrostatic interactions with the negatively charged POPG head groups will be more pronounced than the intramolecular hydrogen bounds formed upon folding into an α-helix.

However, due to the electrostatic interactions between the peptides and POPG, the conjugation-dependency of the triggered membrane destabilization was lost for both JR2KC and CKV_4_. Pure POPG (100 mol%) liposomes gave full or close to full release after addition of JR2KC or CKV_4_, also for the lowest peptide concentrations tested (0.01 and 0.1 μM, respectively) (Fig. S14 and S15A, B, ESI†). This indicates that the high negative net charge of these liposomes was sufficient to localize the peptide close to the lipid bilayer surface. For liposomes with a 1 : 1 ratio of POPC : POPG, still lacking MPB, only the highest peptide concentrations could cause conjugation-independent CF release (Fig. S14 and S15C, D, ESI†). Additionally, DLS measurements indicated major aggregation for these two peptide–liposome combinations, both with and without cholesterol, however only after incubation with JR2KC (Fig. S16 and S17A, B, ESI†). Thus, identical to MPB-containing POPG-liposomes, CKV_4_ did not trigger any lipid vesicle aggregation, as opposed to JR2KC (Fig. S16 and S17C, ESI†). This could again be due to the difference in size and charge between the two peptides. In line with the other observations, presence of cholesterol resulted in a more extensive CF release in POPG/MPB-containing liposomes for all three lipid compositions (POPC:MPB:Chol, POPC:POPG:MPB:Chol and POPG:MPB:Chol) when exposed to JR2KC (Fig. 4A and Fig. S11G–I, ESI†) and extensive liposome aggregation was observed (Fig. 4C and Fig. S13C, ESI†). Cholesterol did not have any influence on the peptide secondary structure and a β-sheet structure was seen also for POPG:MPB:Chol containing liposomes (Fig. 4D). However, cholesterol was found to have a large impact on the membrane activity of JR2KC when interacting with POPG containing liposomes. When excluding the MPB lipid, no CF release was seen for the POPC:POPG:Chol liposome and only minor release at the highest peptide concentrations was seen for POPG:Chol liposomes (Fig. S14A and S15E, F, ESI†). This is in stark contrast to non-cholesterol containing POPG-liposomes lacking MPB where JR2KC caused extensive CF release (Fig. S14A and S15A, E, ESI†). Thus, cholesterol seems to stabilize the lipid membrane enough to re-activate the conjugation-dependance of JR2KC.

## Conclusions

In conclusion, we have demonstrated that the physicochemical properties of liposomes can have a major impact on the function of membrane active peptides designed for triggered release. The effect of liposome size, net charge, cholesterol content and lipid saturation on the membrane activity of the two different *de novo* designed conjugation-dependent membrane active peptides JR2KC and CKV_4_ was explored. Differences in liposome size did not have any major impact on the membrane activity although some changes in secondary structure were observed. A slight increase in CF release was seen for larger lipid vesicles, which most likely was due to a higher number of conjugated peptides per liposome. The effect was more pronounced for the larger and more positively charged peptide JR2KC compared to for CKV_4_. In contrast, a large increase in CF release was seen when combining saturated and unsaturated lipids or when introducing negatively charged lipids in the liposomes. In liposomes with a combination of saturated and unsaturated lipids, the release triggered by conjugation of JR2KC and CKV_4_ was significantly enhanced compared to liposomes with only unsaturated lipids, potentially due to the observed tendency for phase separation, resulting in clustering of the maleimide-headgroup functionalized lipid MPB and thus a clustering of the peptides. A more distinct lipid phase separation was seen in the presence of cholesterol and that was further pronounced upon conjugation of JR2KC, resulting in a dramatic increase in CF release. We have previously seen that JR2KC conjugation can contribute to lipid phase separation in cholesterol-rich lipid vesicles as a result of folding-dependent interactions.^11^ The formation of peptide-rich domains can likely increase the line tension at membrane domain boundaries and facilitate the partitioning of the peptide into the lipid membrane. Not surprisingly, however, the factor that had the largest influence on liposomes stability was lipid net charge. By including the highly negatively charged lipid POPG in the liposome formulation, the electrostatically driven adsorption of the peptides at the lipid membrane resulted in pronounced CF release also in the absence of MPB. Indirectly, this finding further confirms that in liposomes without POPG, the conjugation of the peptides increases the surface concentration and residence time of the peptides at the lipid membrane, lowering the energy barrier for membrane partitioning. Increasing the POPG content had a more dramatic impact on the function of JR2KC compared to CKV_4_, likely due to its larger size and higher positive net charge, which resulted in differences in both peptide secondary structure and colloidal stability of the liposomes. Based on the findings described herein, we can better guide the design of MAP-triggered liposomal drug delivery systems, where all aspects of the close interplay between peptide primary structure and lipid membrane properties must be considered to ensure liposome stability and enable tuning of drug release using specific peptide mediated interactions.

## Conflicts of interest

There are no conflicts of interest to declare.

## Supplementary Material

TB-012-D4TB01107D-s001

## Data Availability

Data for this article, including CF release data, CD spectra, and DLS data, are available at Zenodo at https://doi.org/10.5281/zenodo.13332865.
