# Supplementary material for: Influence of lipid vesicle properties on the function of conjugation dependent membrane active peptides
Source: J Mater Chem B. 2024 Sep 5;12(40):10320–31. doi: 10.1039/d4tb01107d (PMC11409839; doi:10.1039/d4tb01107d)
Supplement: TB-012-D4TB01107D-s001 [file TB-012-D4TB01107D-s001.pdf]

Supplementary Information (SI) for Journal of Materials Chemistry B.
This journal is © The Royal Society of Chemistry 2024

Supporting Information

Influence of Lipid Vesicle Properties on the Function of Conjugation Dependent Membrane Active Peptides

*Alexandra Iversen,† Johanna Utterström,† Lalit Pramod Khare, Daniel Aili**

Laboratory of Molecular Materials, Division of Biophysics and Bioengineering, Department of Physics, Chemistry, and Biology, Linköping University, 581 83 Linköping, Sweden.

† *Equal contributions*

* Corresponding author: daniel.aili@liu.se

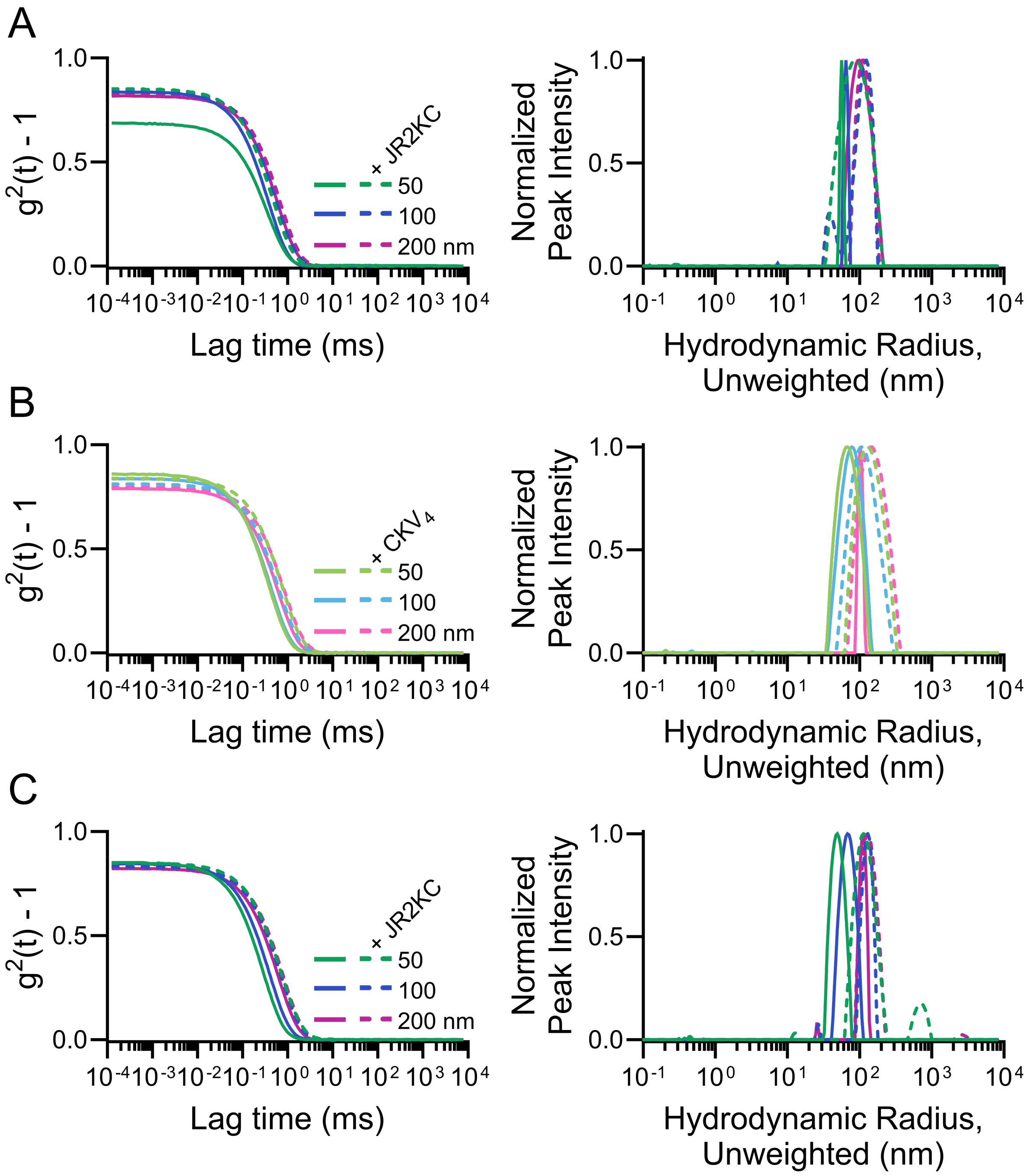

Figure S1. DLS correlation function and size distribution of 40 µM 50, 100, and 200 nm A) 95:5 POPC:MPB liposomes before and after 30 min incubation with 4 µM JR2KC. B) 95:5 POPC:MPB liposomes before and after 2 hours incubation with 10 µM CKV_4. C) 65:5:30 POPC:MPB:Chol liposomes before and after 30 min incubation with 4 µM JR2KC.

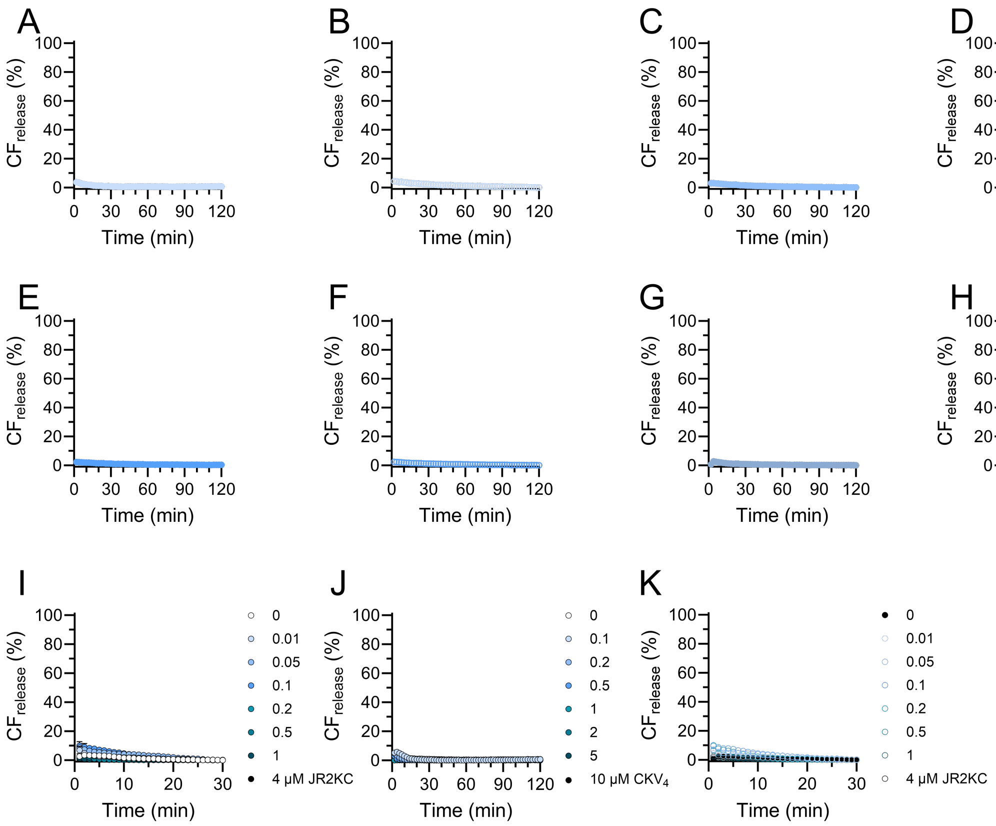

Figure S2. CF release kinetics during 2 hours from 40 µM A) 95:5:0 50 nm, B) 65:5:30 50 nm, C) 95:5:0 100 nm, D) 65:5:30 100 nm, E) 95:5:0 200 nm, F) 65:5:30 200 nm, G) 100:0:0 100 nm, H) 70:0:30 100 nm POPC:MPB:Chol liposomes, I-J) 100:0:0 100 nm POPC:MPB:Chol liposomes incubated with various concentrations of I) JR2KC, or J) CKV_4, and K) 70:0:30 100 nm POPC:MPB:Chol liposomes incubated with various concentrations of JR2KC.

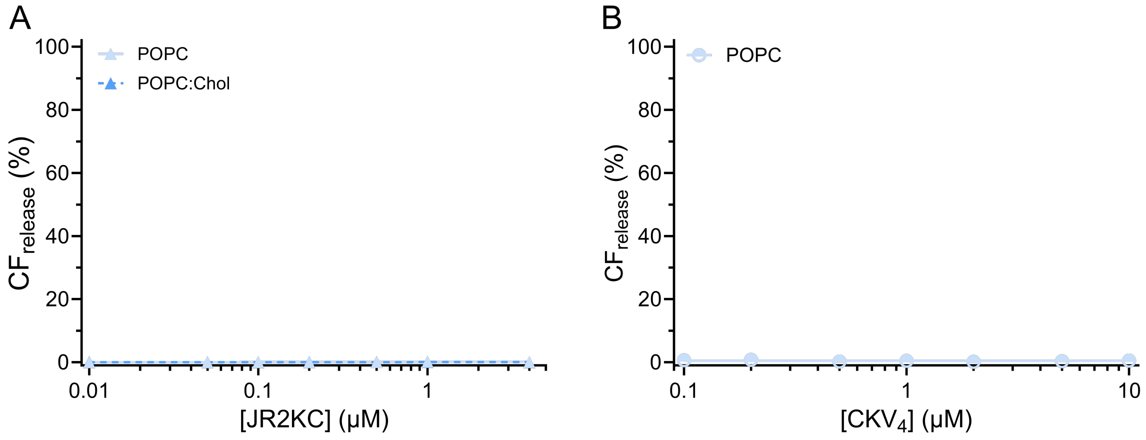

Figure S3. A) Total CF release from 40 µM 100 nm 100:0, 70:30 POPC:Chol liposomes after 30 min incubation with JR2KC. B) Total CF release from 40 µM 100 nm 100 mol% POPC liposomes after 2 hours incubation with CKV_4.

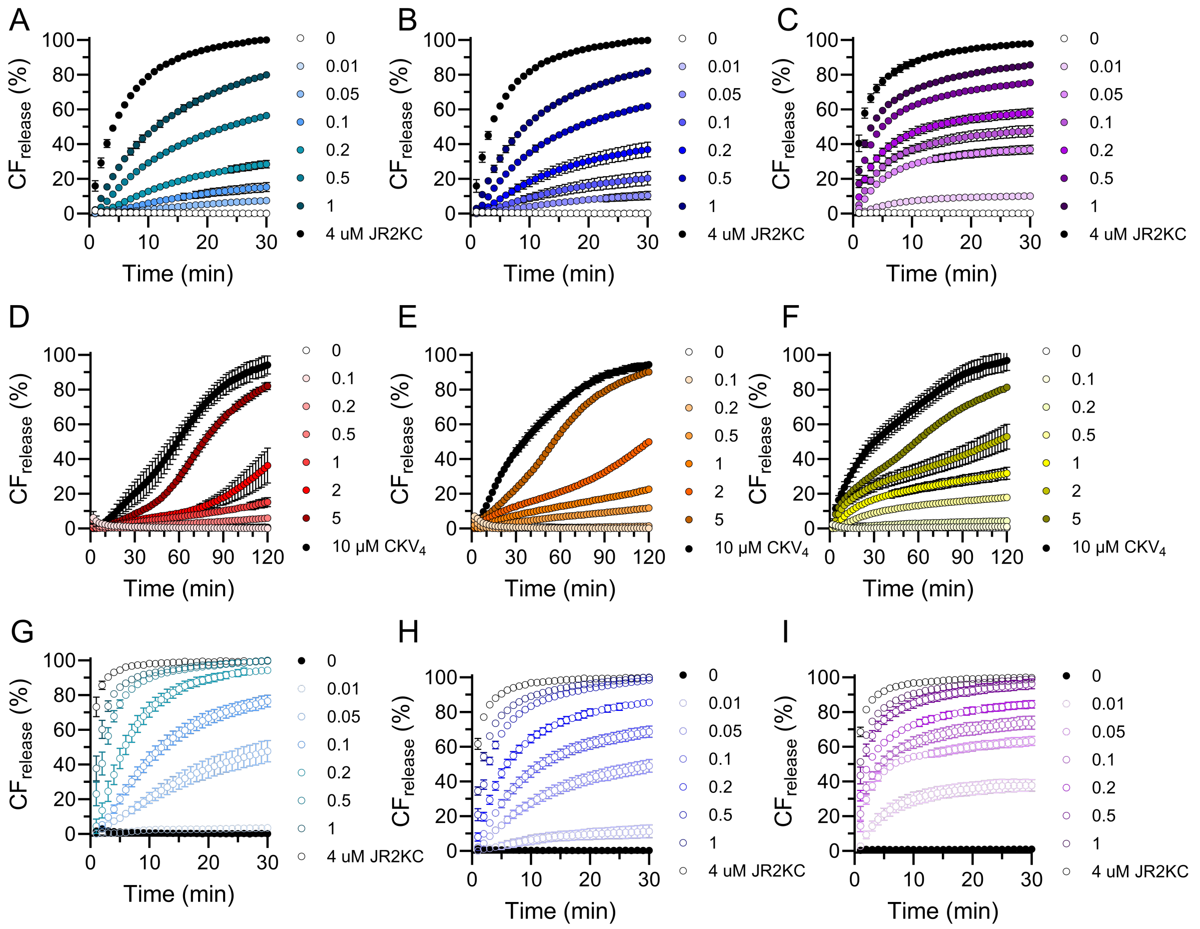

Figure S4. A-C) CF release kinetics during 30 min incubation with varying JR2KC concentrations and 40 µM A) 50 nm, B) 100 nm, C) 200 nm 95:5 POPC:MPB liposomes. D-F) CF release kinetics during 2 hours incubation with varying CKV_4 concentrations and 40 µM D) 50 nm, E) 100 nm, F) 200 nm 95:5 POPC:MPB liposomes. G-I) CF release kinetics during 30 min incubation with varying JR2KC concentrations and 40 µM G) 50 nm, H) 100 nm, I) 200 nm 65:5:30 POPC:MPB:Chol liposomes.

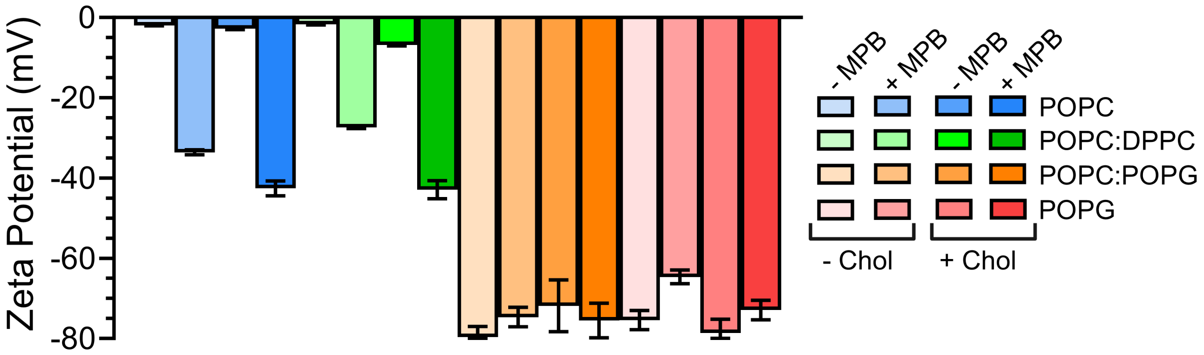

Figure S5. Zeta potential of liposomes with varying mol% lipids with saturated acyl chains and negatively charged headgroup, with and without MPB and cholesterol.

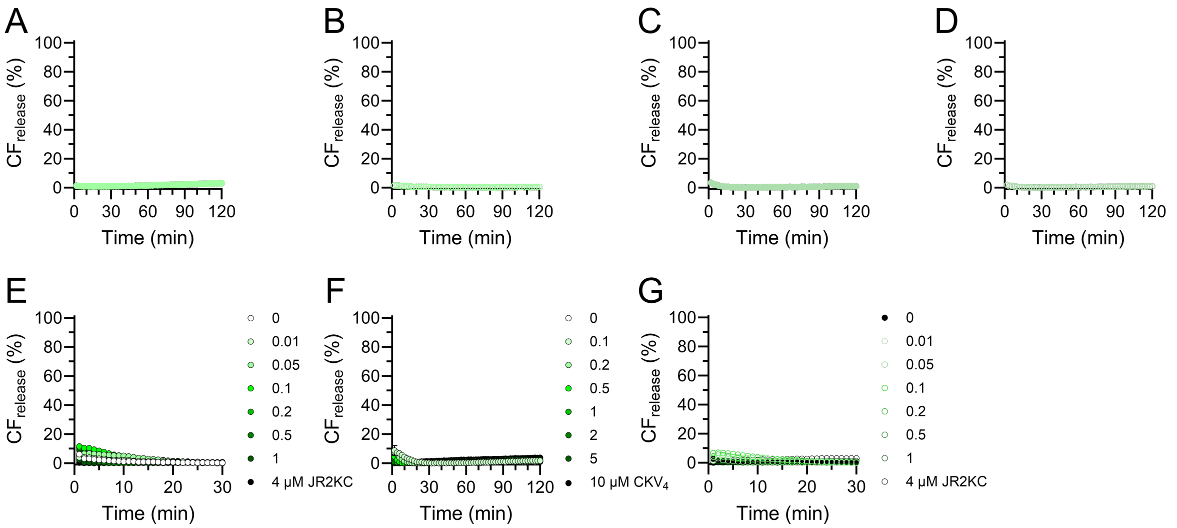

Figure S6. CF release kinetics during 2 hours from 40 μM A) 47.5:47.5:5:0, B) 32.5:32.5:5:30, C) 50:50:0:0, D) 35:35:0:30 100 nm POPC:DPPC:MPB:Chol liposomes. E-F) 50:50:0:0 POPC:DPPC:MPB:Chol liposomes incubated with various concentrations of E) JR2KC, or F) CKV_4, and G) 35:35:0:30 POPC:DPPC:MPB:Chol liposomes incubated with various concentrations of JR2KC.

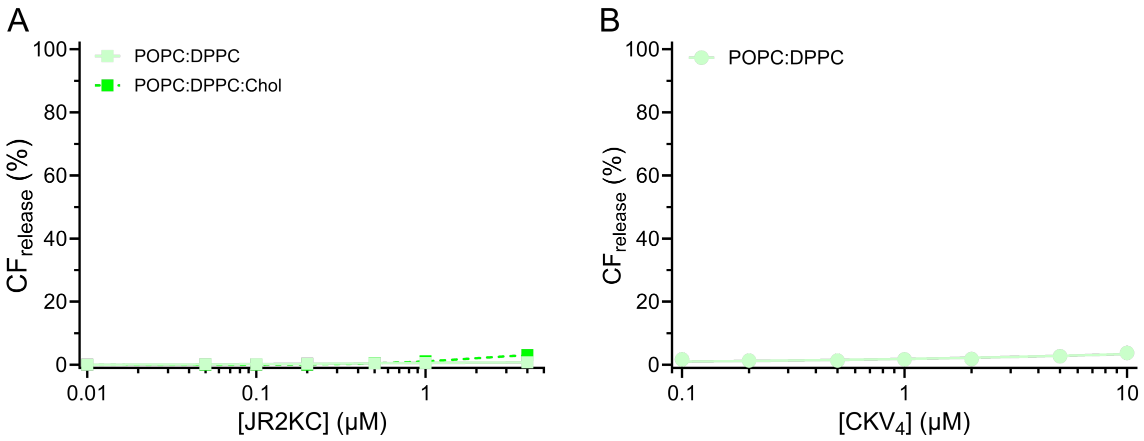

Figure S7. A) Total CF release from 40 μM 50:50:0, 35:35:30 POPC:DPPC:Chol liposomes after 30 min incubation with JR2KC. B) Total CF release from 40 μM 50:50 POPC:DPPC liposomes after 2 hours incubation with CKV_4.

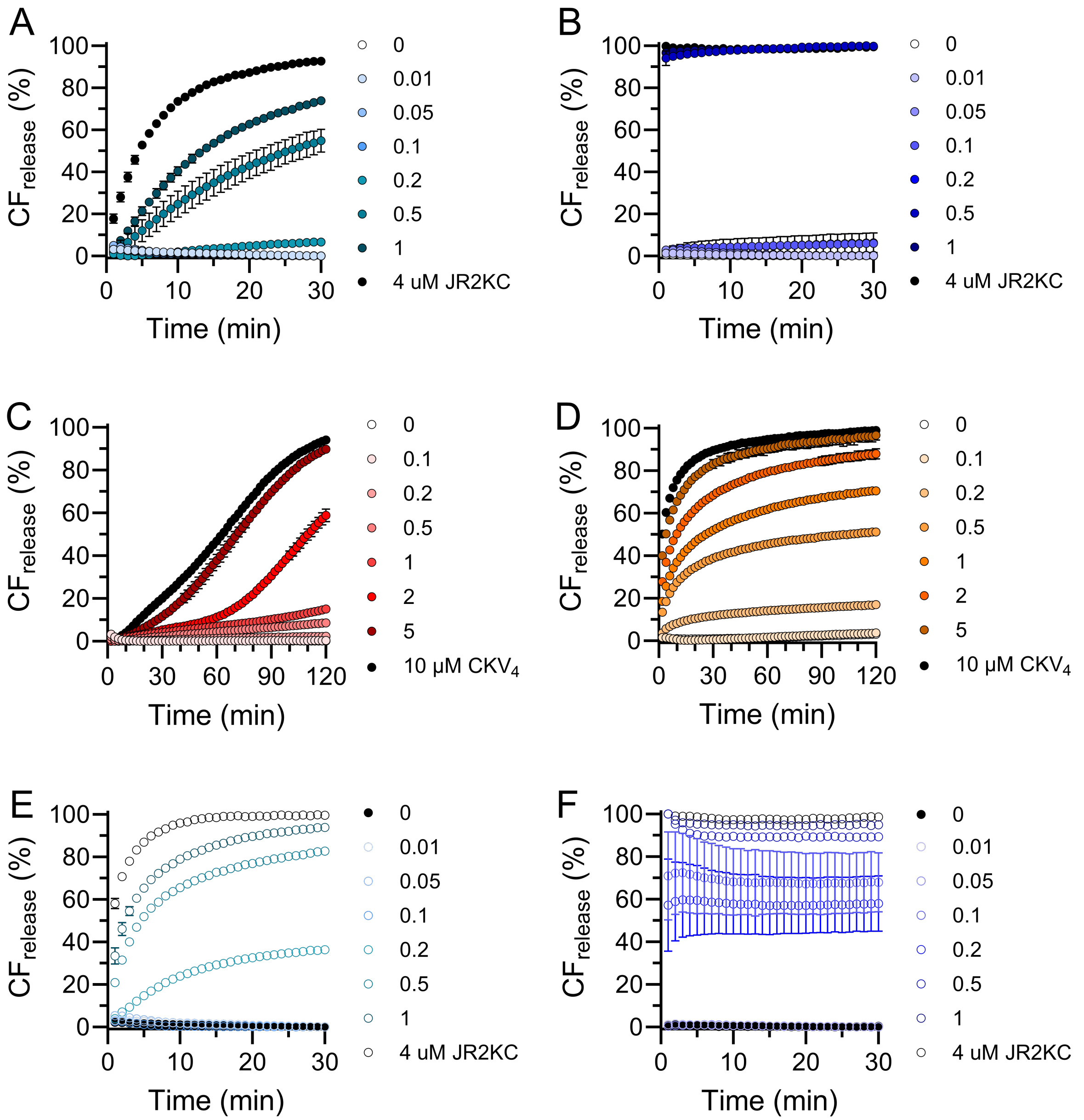

Figure S8. A-B) CF release kinetics during 30 min incubation with varying JR2KC concentrations and 40 µM A) 95:0:5, B) 47.5:47.5:5 POPC:DPPC:MPB liposomes. C-D) CF release kinetics during 2 hours incubation with varying CKV_4 concentrations and C) 95:0:5, D) 47.5:47.5:5 POPC:DPPC:MPB liposomes. E-F) CF release kinetics during 30 min incubation with varying JR2KC concentrations and E) 65:0:5:30, F) 32.5:32.5:5:30 POPC:DPPC:MPB:Chol liposomes.

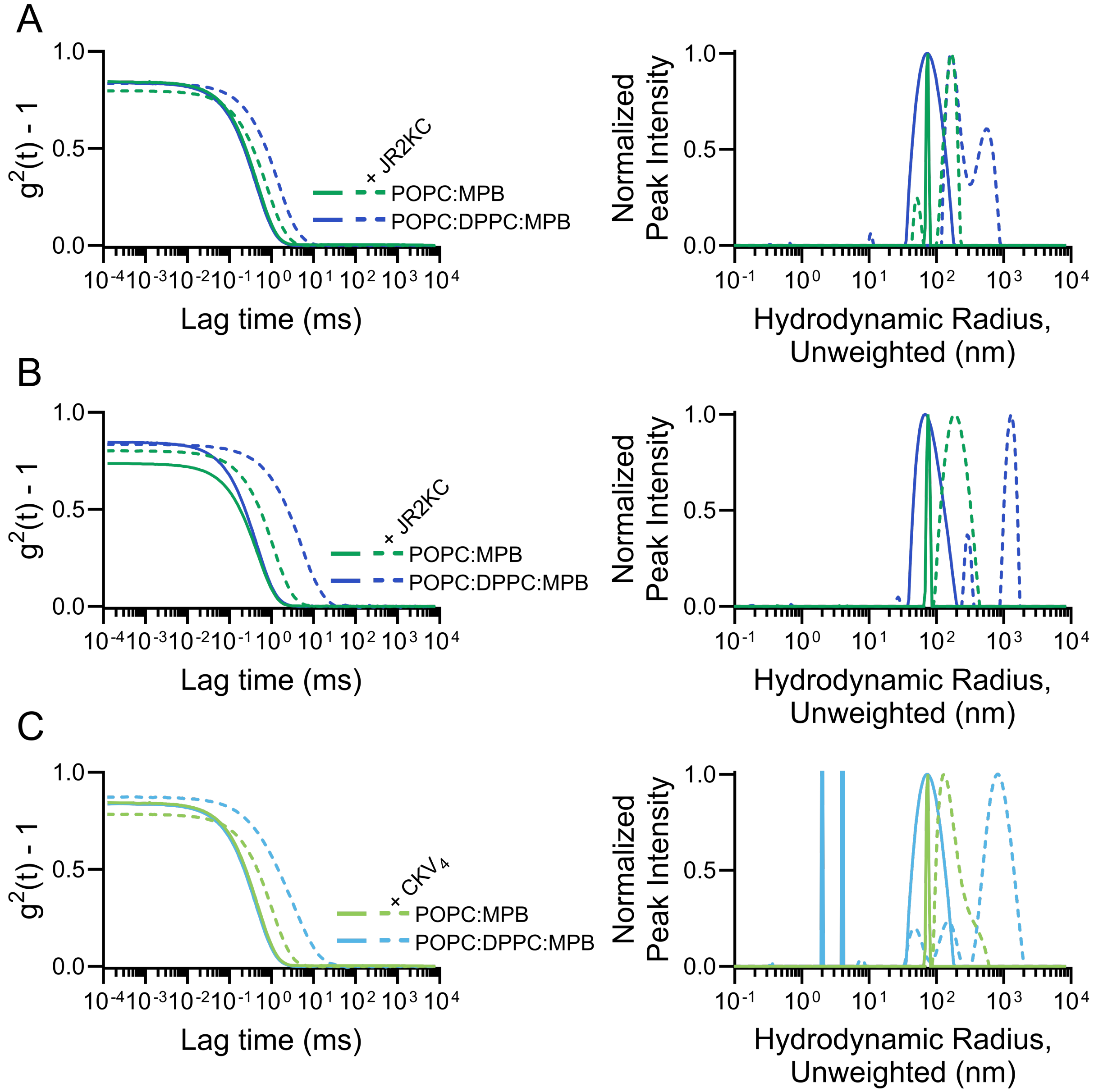

Figure S9. A) DLS correlation function and size distribution of 40 µM 95:0:5 and 47.5:47.5:5 POPC:DPPC:MPB liposomes before and after 30 min incubation with 4 µM JR2KC. B) DLS correlation function and size distribution of 40 µM 65:0:5:30 and 32.5:32.5:5:30 POPC:DPPC:MPB:Chol liposomes before and after 30 min incubation with 4 µM JR2KC. C) DLS correlation function and size distribution of 40 µM 95:0:5 and 47.5:47.5:5 POPC:DPPC:MPB liposomes before and after 2 hours incubation with 10 µM CKV$_4$.

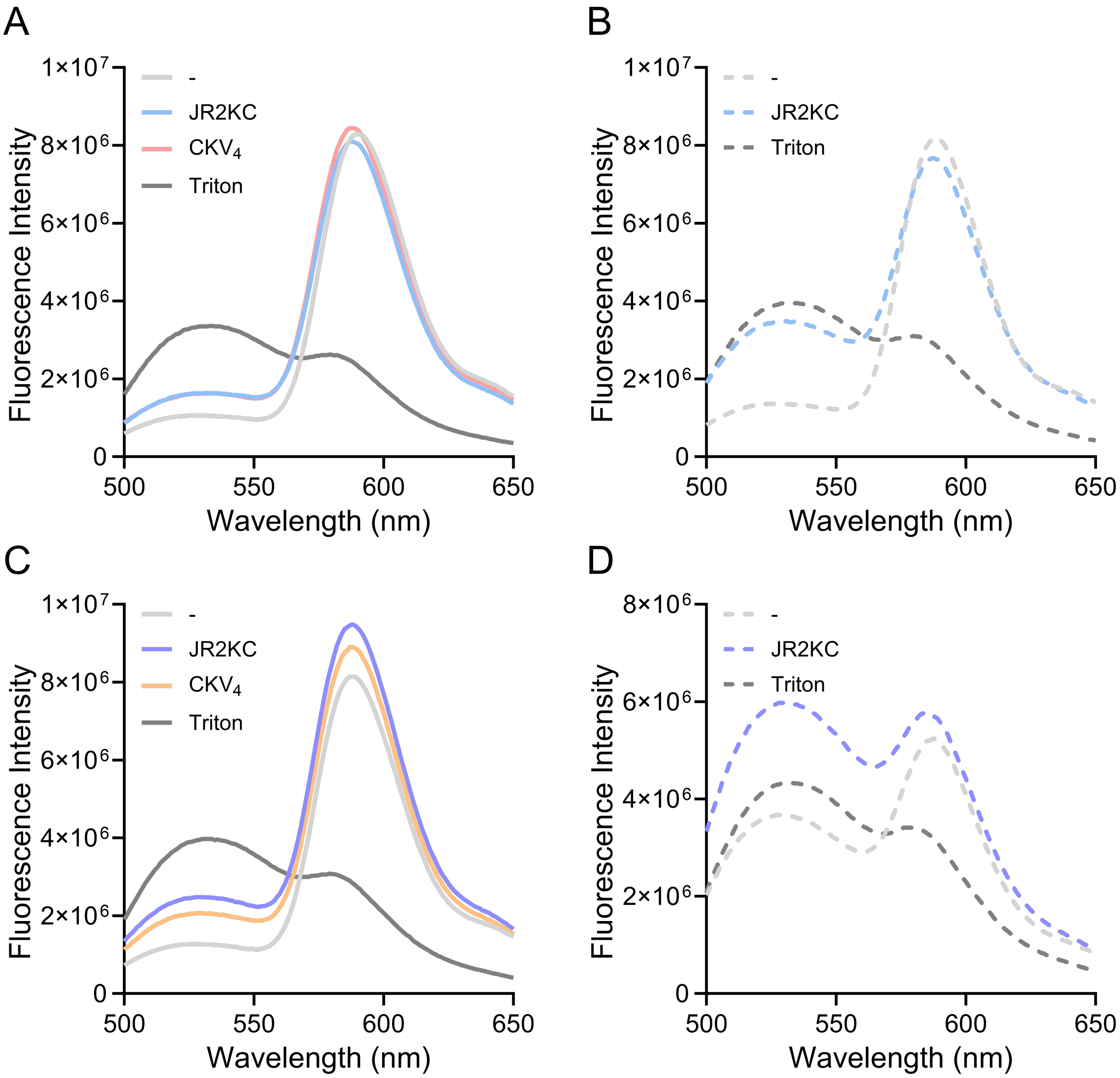

Figure S10. FRET wavelength scan of 40 µM A) 94:0:5:0:0.5:0.5, B) 64:0:5:30:0.5:0.5, C) 47:47:5:0:0.5:0.5, D) 32:32:5:30:0.5:0.5 POPC:DPPC:MPB:Chol:Rh:NBD liposomes before and after 30 min incubation with 4 µM JR2KC or 2 hours incubation with 10 µM CKV_4 respectively and after addition of triton.

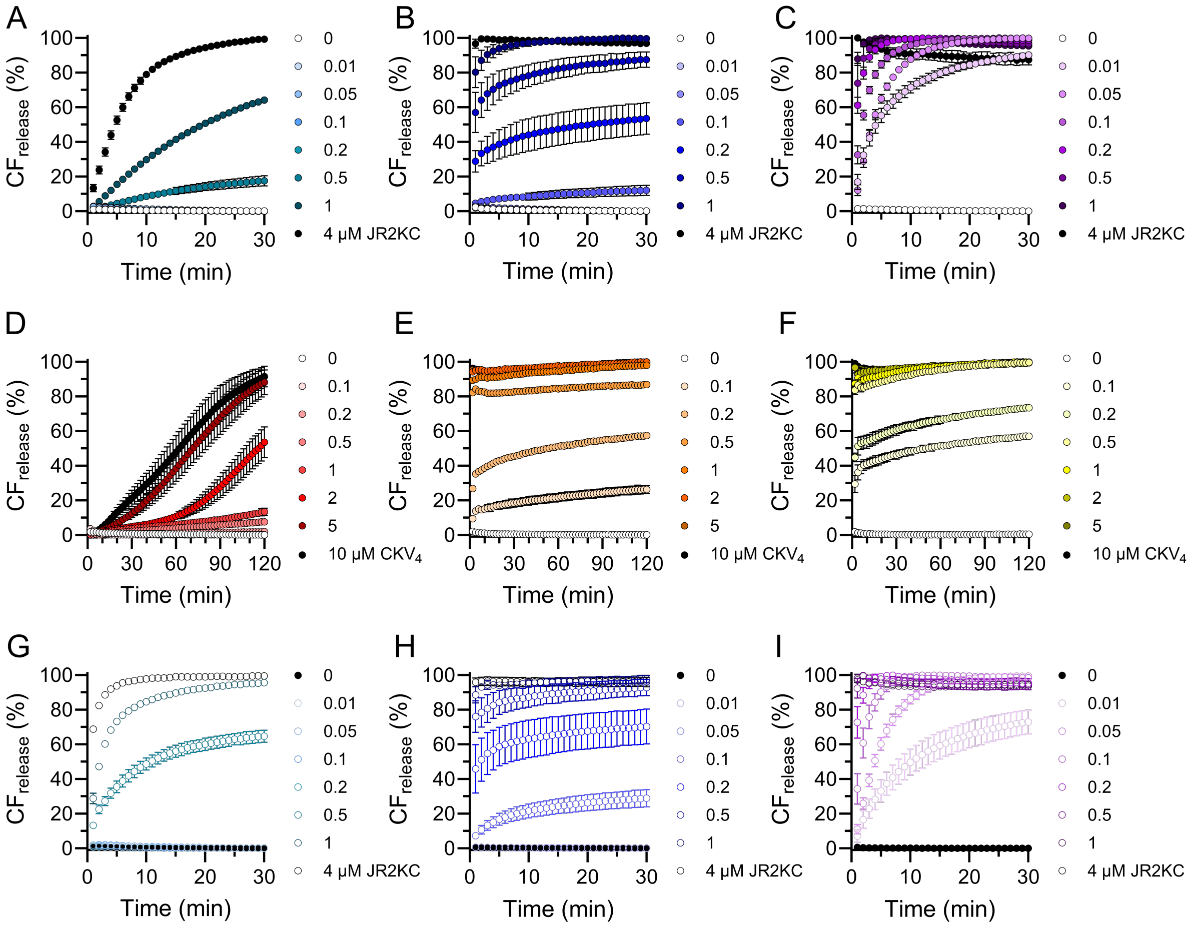

Figure S11. A-C) CF release kinetics during 30 min incubation with varying JR2KC concentrations and 40 µM A) 95:0:5, B) 47.5:47.5:5, C) 0:95:5 POPC:POPG:MPB liposomes. D-F) CF release kinetics during 2 hours incubation with varying CKV$_4$ concentrations and 40 µM D) 95:0:5, E) 47.5:47.5:5, F) 0:95:5 POPC:POPG:MPB liposomes. G-I) CF release kinetics during 30 min incubation with varying JR2KC concentrations and 40 µM G) 65:0:5:30, H) 32.5:32.5:5:30, I) 0:65:5:30 POPC:POPG:MPB:Chol liposomes.

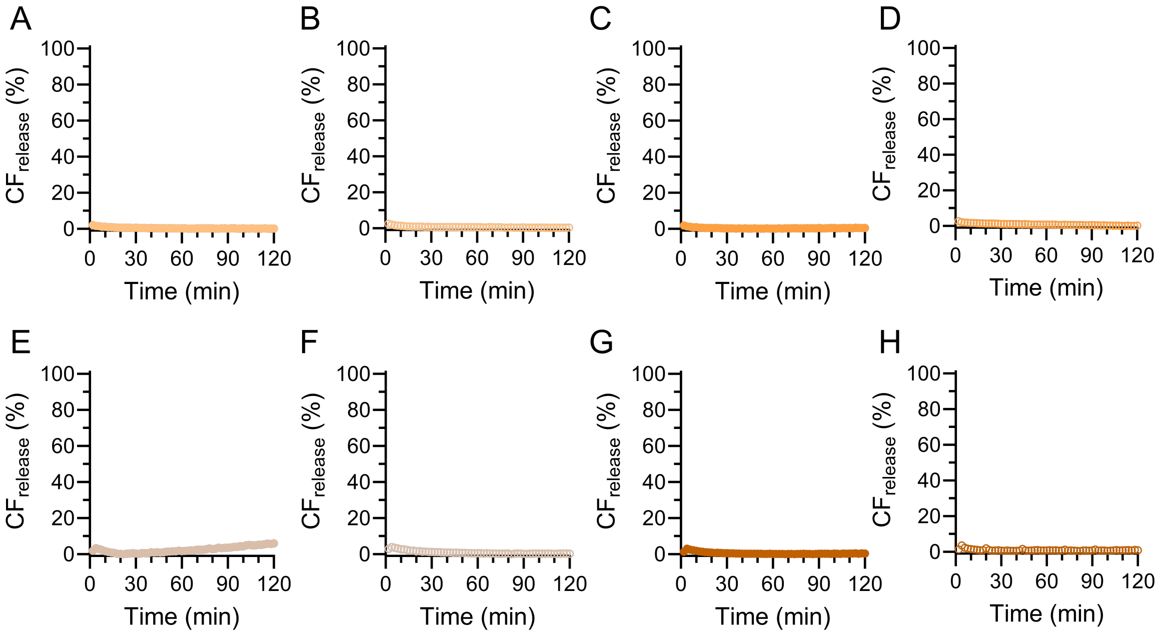

Figure S12. CF release kinetics during 2 hours from 40 µM A) 47.5:47.5:5:0, B) 32.5:32.5:5:30, C) 0:95:5:0, D) 0:65:5:30, E) 50:50:0:0, F) 35:35:0:30, G) 0:100:0:0, H) 0:70:0:30 100 nm POPC:DPPC:POPG:MPB:Chol liposomes.

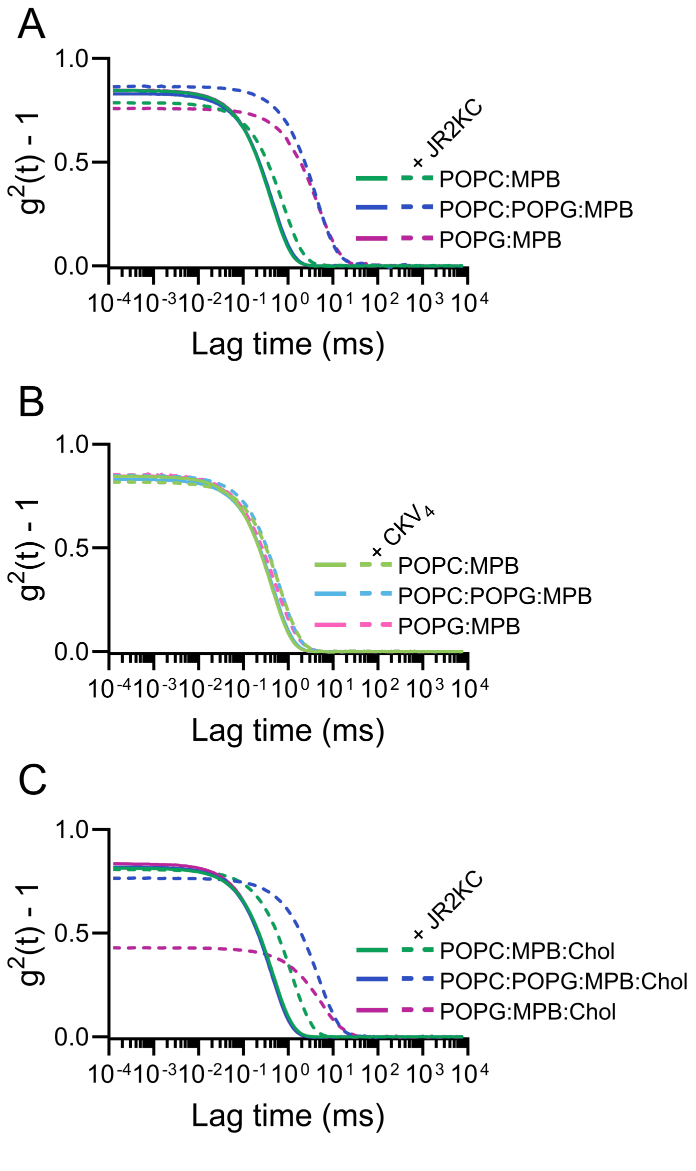

Figure S13. A) DLS correlation function and size distribution of 40 µM 95:0:5, 47.5:47.5:5, and 0:95:5 POPC:POPG:MPB liposomes before and after 30 min incubation with 4 µM JR2KC. B) DLS correlation function and size distribution of 40 µM 95:0:5, 47.5:47.5:5, and 0:95:5 POPC:POPG:MPB liposomes before and after 2 hours incubation with 10 µM CKV_4. C) DLS correlation function and size distribution of 40 µM 65:0:5:30, 32.5:32.5:5:30, and 0:65:5:30 POPC:POPG:MPB:Chol liposomes before and after 30 min incubation with 4 µM JR2KC.

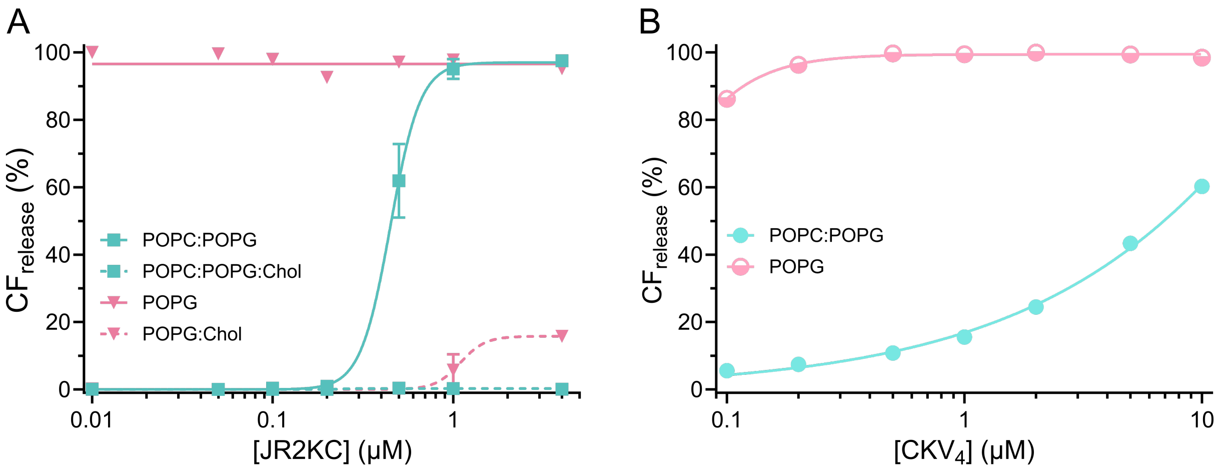

Figure S14. A) Total CF release from 40 μM 50:50:0, 35:35:30, 0:100:0, 0:70:30 POPC:POPG:Chol liposomes after 30 min incubation with JR2KC. B) Total CF release from 40 μM 50:50, and 0:100 POPC:POPG liposomes after 2 hours incubation with CKV_4.

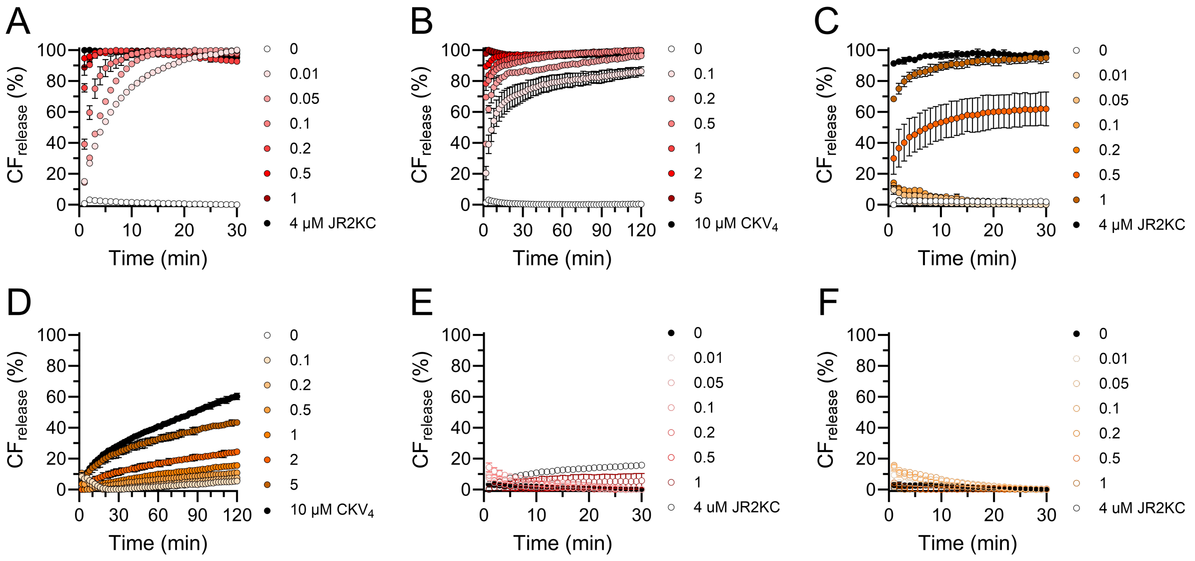

Figure S15. CF release kinetics from 40 μM A-B) 100 mol% POPG liposomes over A) 30 min incubation with JR2KC, or B) 2 hours incubation with CKV_4. C-D) 50:50 POPC:POPG liposomes over C) 30 min incubation with JR2KC, or D) 2 hours incubation with CKV_4. E) 70:30 POPG:Chol liposomes over 30 min incubation with JR2KC. F) 35:35:30 POPC:POPG:Chol liposomes over 30 min incubation with JR2KC.

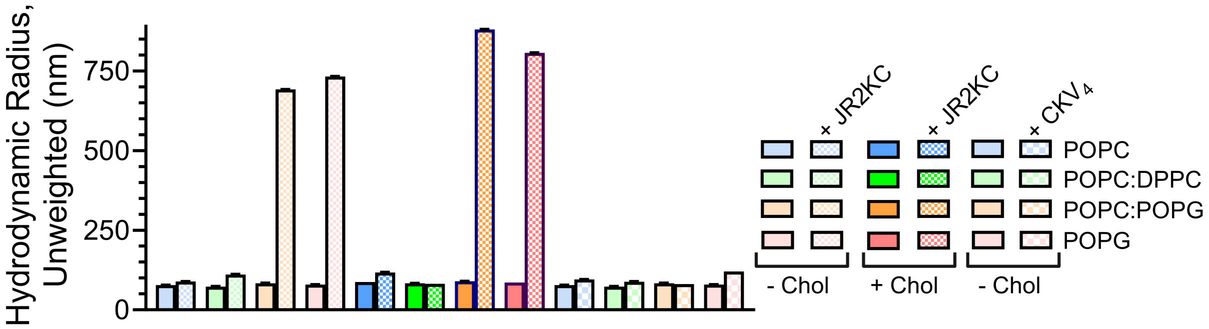

Figure S16. Hydrodynamic radius of 40 µM liposomes with varying mol% lipids with saturated acyl chains and negatively charged headgroup before and after 30 min incubation with 4 µM JR2KC or 2 hours incubation with 10 µM CKV_4 respectively.

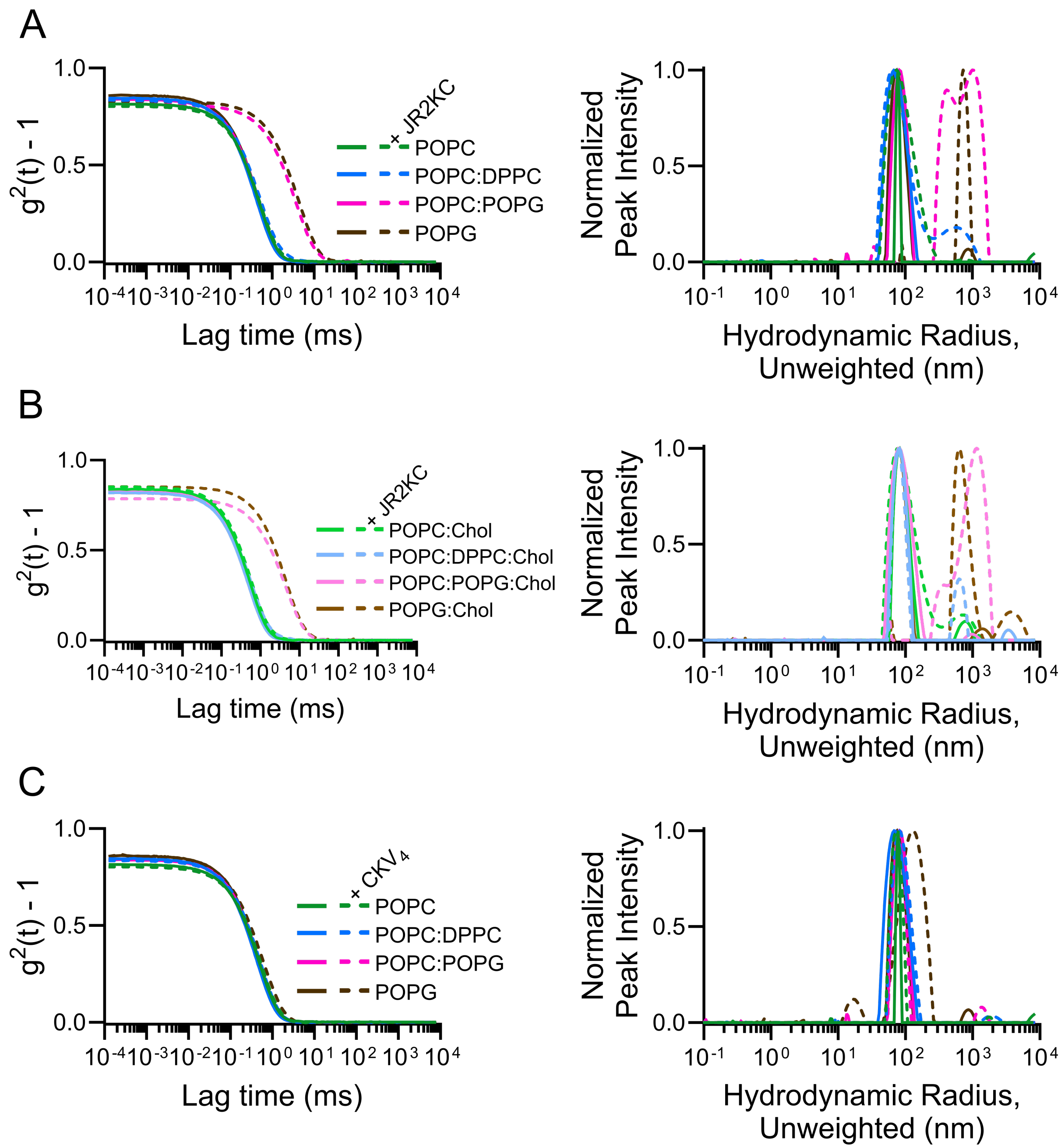

Figure S17. A) DLS correlation function and size distribution of 40 μM 100:0:0, 50:50:0, 50:0:50 and 0:0:100 POPC:DPPC:POPG liposomes before and after 30 min incubation with 4 μM JR2KC. B) DLS correlation function and size distribution of 40 μM 70:0:0:30, 35:35:0:30, 35:0:35:30, and 0:0:70:30 POPC:DPPC:POPG:Chol liposomes before and after 30 min incubation with 4 μM JR2KC. C) DLS correlation function and size distribution of 40 μM 100:0:0, 50:50:0, 50:0:50 and 0:0:100 POPC:DPPC:POPG liposomes before and after 2 hours incubation with 10 μM CKV_4.
